# Target Abundance-Based Fitness Screening (TAFiS) Facilitates Rapid Identification of Target-Specific and Physiologically Active Chemical Probes

**DOI:** 10.1128/mSphere.00379-17

**Published:** 2017-10-04

**Authors:** Arielle Butts, Christian DeJarnette, Tracy L. Peters, Josie E. Parker, Morgan E. Kerns, Karen E. Eberle, Steve L. Kelly, Glen E. Palmer

**Affiliations:** a Department of Clinical Pharmacy and Translational Science, College of Pharmacy, University of Tennessee Health Sciences Center, Memphis, Tennessee, USA; b Department of Molecular Immunology and Biochemistry, College of Graduate Health Sciences, University of Tennessee Health Sciences Center, Memphis, Tennessee, USA; c Institute of Life Science, Swansea University Medical School, Swansea, Wales, United Kingdom; d Department of Microbiology, Immunology, and Parasitology, School of Medicine, Louisiana State University Health Sciences Center, New Orleans, Louisiana, USA; Duke University Medical Center

**Keywords:** *Candida albicans*, antifungal agents, chemical genetics, drug screening

## Abstract

Traditional approaches to drug discovery are frustratingly inefficient and have several key limitations that severely constrain our capacity to rapidly identify and develop novel experimental therapeutics. To address this, we have devised a second-generation target-based whole-cell screening assay based on the principles of competitive fitness, which can rapidly identify target-specific and physiologically active compounds. Briefly, strains expressing high, intermediate, and low levels of a preselected target protein are constructed, tagged with spectrally distinct fluorescent proteins (FPs), and pooled. The pooled strains are then grown in the presence of various small molecules, and the relative growth of each strain within the mixed culture is compared by measuring the intensity of the corresponding FP tags. Chemical-induced population shifts indicate that the bioactivity of a small molecule is dependent upon the target protein’s abundance and thus establish a specific functional interaction. Here, we describe the molecular tools required to apply this technique in the prevalent human fungal pathogen *Candida albicans* and validate the approach using two well-characterized drug targets—lanosterol demethylase and dihydrofolate reductase. However, our approach, which we have termed target abundance-based fitness screening (TAFiS), should be applicable to a wide array of molecular targets and in essentially any genetically tractable microbe.

**IMPORTANCE** Conventional drug screening typically employs either target-based or cell-based approaches. The first group relies on biochemical assays to detect modulators of a purified target. However, hits frequently lack drug-like characteristics such as membrane permeability and target specificity. Cell-based screens identify compounds that induce a desired phenotype, but the target is unknown, which severely restricts further development and optimization. To address these issues, we have developed a second-generation target-based whole-cell screening approach that incorporates the principles of both chemical genetics and competitive fitness, which enables the identification of target-specific and physiologically active compounds from a single screen. We have chosen to validate this approach using the important human fungal pathogen *Candida albicans* with the intention of pursuing novel antifungal targets. However, this approach is broadly applicable and is expected to dramatically reduce the time and resources required to progress from screening hit to lead compound.

## Abstract (precis)

Conventional drug screening typically employs either target-based or cell-based approaches. The first group rely on biochemical assays to detect modulators of a purified target. However, hits frequently lack drug-like characteristics such as membrane permeability and target specificity. Cell-based screens identify compounds that induce a desired phenotype, but the target is unknown, which severely restricts further development and optimization. To address these issues, we have developed a second-generation target-based whole-cell screening approach that incorporates the principles of both chemical genetics and competitive fitness, which enables the identification of target-specific and physiologically active compounds from a single screen. We have chosen to validate this approach using the important human fungal pathogen *Candida albicans* with the intention of pursuing novel antifungal targets. However, this approach is broadly applicable and is expected to dramatically reduce the time and resources required to progress from screening hit to lead compound.

## INTRODUCTION

The process of drug discovery, development, and approval often takes more than a decade, with a recent report estimating the associated costs at over $2.5 billion (1). As such, there is an urgent need to increase the efficiency with which new experimental therapeutics are discovered and developed. This is especially true for antifungal development, with the newest class of antifungal drugs, the echinocandins, taking 30 years to reach patients (2). High attrition rates at both the preclinical and clinical stages prolong this timeline and greatly increase the associated costs. Many failures occur because hit compounds selected from the initial chemical screening steps lack the qualities of an effective therapy, such as target specificity or membrane permeability. This is a direct result of the significant limitations of the two approaches most widely adopted to seek chemicals with the desired activity. Target-based screens require the development of a biochemical assay that is utilized to identify inhibitors or activators of a purified target protein. While this is an effective strategy to identify potent chemical modulators of the target protein, many have poor membrane permeability or do not otherwise engage the target protein in its native environment. As such, many hits from target-based screens lack activity upon whole cells or are later found to lack target specificity and consequently have unintended off-target effects and/or toxicity. This necessitates costly efforts to improve cell permeability and/or selective target engagement that are often unsuccessful. Thus, hits obtained through target-based screens have not translated well into clinically useful antimicrobial agents (3). Furthermore, many proteins are not amenable to purification or high-throughput (HTP)-compatible biochemical assays and thus are not suited for target-based chemical screens. Whole-cell-based screens identify compounds that induce or correct a disease-relevant phenotype (e.g., inhibition of microbial growth), but the molecular target and mechanism of action (MOA) of each hit are unknown. Identification of the molecular target then requires a substantial investment of time and resources, without which further development and optimization of promising lead compounds toward a viable therapeutic is severely restricted (4, 5). Thus, with either strategy, the identification of pharmacologically active agents that act via a defined MOA is a multistep process. Significant increases in the efficiency of drug discovery and development can be achieved through the rapid identification of physiologically active hits that act upon a specific target protein or pathway within living cells. Furthermore, the elimination of agents that lack the requisite target specificity during primary screening would also yield dramatic cost and time savings.

The purpose of this study was to establish and validate an innovative HTP screening strategy that can dramatically improve the efficiency with which target-specific and physiologically active chemical probes are identified. Our approach is a type of target-based whole-cell screen (TB-WCS), which refers to the identification of chemical probes that functionally interact with a selected target protein within intact cells (6). Like previous TB-WCS screens, our method, which we have termed target abundance-based fitness screening (TAFiS), relies on the fundamental principles of chemical genetics—that altering the abundance of a target protein usually affects a cell’s susceptibility to chemical modulators of that target (7). However, TAFiS also integrates the principle of competitive fitness to enhance the efficiency and sensitivity of the screening assay and incorporates fluorescent protein (FP) tags to facilitate quantitative measurement of the chemical-target interaction. To provide proof of principle, we have developed the tools and methodology necessary to conduct TAFiS in the prevalent human fungal pathogen *Candida albicans*. We also validated TAFiS using two well-characterized drug targets: lanosterol demethylase (Erg11p)—the target of the azole antifungals—and dihydrofolate reductase (Dfr1p). However, the approach we have developed is broadly applicable and can theoretically be applied to almost any target and in any genetically tractable microbe.

## RESULTS

### Design of TAFiS assay.

**FIG 1:**
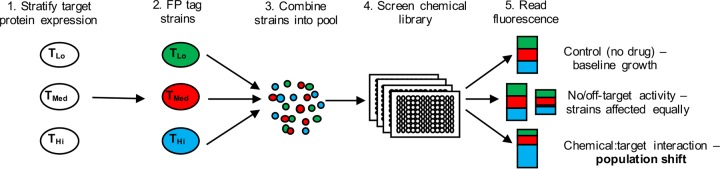
Schematic of the target abundance-based fitness screening (TAFiS) assay. A panel of strains expressing high (T_Hi_), medium (T_Med_), or low (T_Lo_) levels of the selected target are constructed, tagged with spectrally distinct fluorescent proteins (FP), and pooled. The expression pool is then incubated in the presence of various small molecules, and the relative growth of each strain is quantified by measuring fluorescence. On-target inhibitors should differentially impact the growth of each strain causing a chemical-induced population shift that can be detected as a shift in fluorescence compared to the untreated control.

Strains expressing high (T_Hi_), low (T_Lo_), or intermediate (T_Med_) levels of the desired target protein are constructed, and each is labeled with a spectrally distinct fluorescent protein (FP) tag. The tagged strains are then mixed together to form an expression pool that is used to screen compounds and identify those that differentially affect the growth of each strain. The resulting chemical-induced population shift can be detected spectroscopically and indicates that a compound’s bioactivity depends upon the abundance of the target protein—therefore establishing a functional chemical-target interaction (Fig. 1). As such, TAFiS depends upon two key technical challenges: (i) the capacity to label individual *C. albicans* strains with sufficiently bright and spectrally distinct FP tags and (ii) the stratification of target protein expression between T_Hi_, T_Med_, and T_Lo_ strains.

### Selection and optimization of fluorescent protein tags.

The coding sequences of several previously reported FPs (8–15) were cloned into expression vectors, which were then stably integrated into the genome of *C. albicans*, and the fluorescence intensity of each FP-tagged strain was compared to that of an untagged strain transformed with vector alone. The strains were grown in 96-well plates in yeast nitrogen base (YNB) medium at 30°C for 48 h to mimic the proposed conditions of the chemical screen, before fluorescence intensity was measured at the reported excitation and emission maxima of each FP. Of those tested, the three brightest FP tags under the proposed assay conditions were cerulean (CER), green fluorescent protein gamma (GFPγ), and dTomato (dTOM) (see Fig. S1A in the supplemental material). We next examined the degree of cross-excitation between these three FP tags by measuring the fluorescence signal of each tagged strain at each of the FP wavelengths. Minimal spectral overlap was detected between CER-, GFPγ-, and dTOM-tagged strains (Fig. S1B), and these three FPs were therefore used in subsequent work. We next confirmed that the relative abundance of each tagged strain within a mixed culture can be accurately quantified through fluorescence detection. Three wild-type *C. albicans* strains tagged with each FP were mixed in defined ratios and grown in 96-well plates. After 24 and 48 h, the fluorescence intensity of each FP tag was measured and plotted against the original inoculum. This produced a linear correlation with excellent *R*^2^ values for each FP-tagged strain (Fig. S1C). Finally, we examined if high-level expression of CER, GFPγ, or dTOM was detrimental to *C. albicans* fitness by comparing the capacity of each tagged strain to endure a variety of stresses to that of an untagged control strain, including elevated temperature, osmotic and ionic stresses, as well as the presence of cell wall- and membrane-perturbing agents. While the FP-tagged strains grew marginally slower than the untagged control strain under standard culture conditions (presumably as a result of the metabolic load of FP production), the effect was of similar magnitude irrespective of the FP tag expressed. The FP-tagged strains did not exhibit any additional abnormal phenotypes under any of the stress conditions tested (see Fig. S2 in the supplemental material).

10.1128/mSphere.00379-17.2 FIG S1  The relative growth of each *Candida albicans* strain within a mixed culture can be accurately measured using spectrally distinct fluorescent protein tags. (A) Fluorescence was read at the optimal excitation/emission wavelengths reported for five transformants with each FP. Brightness is expressed as fold change over an untagged control strain. (B) The fluorescence of the brightest transformants expressing CER, GFPγ, and dTOM was read at all three FP wavelengths (excitation/emission of 433/475, 488/507, and 554/581) with a 9-nm band-pass filter to determine the extent of spectral overlap. The mean and standard deviations for each are presented. (C) The brightest strains expressing CER, GFPγ, or dTOM were mixed in defined ratios and grown for 48 h before fluorescence intensity was measured. The percentage of fluorescence at each FP’s wavelength was then plotted against the percentage of that tagged strain in the initial inoculum, resulting in linear correlations with excellent *R*^2^ values for each tag. The values presented are means and standard deviations. Download FIG S1, PDF file, 0.2 MB. Copyright © 2017 Butts et al. 2017 Butts et al. This content is distributed under the terms of the Creative Commons Attribution 4.0 International license.

10.1128/mSphere.00379-17.3 FIG S2  High-level expression of the selected fluorescent proteins does not result in differential susceptibility to a variety of stress. The brightest transformants expressing CER, GFPγ, φYFP, and dTOM were grown overnight in YPD at 30°C. Cells were washed and resuspended in sterile water at approximately 10^7^ cells/ml. Tenfold serial dilutions were pinned on various media in duplicate and incubated for 48 h at 30°C, except when indicated otherwise, prior to imaging. The conditions tested were YPD, YPD at 37°C, YPD at 42°C, YPD plus 1 M sorbitol, YPD plus 1 M NaCl, YPD plus 50 µM CuCl_2_, YPD plus 50 µM ZnCl_2_, YPD plus 10 mM MnCl_2_, YPD plus 5 mM caffeine, YPD plus 25 µg/ml Congo red, YPD plus 0.05% SDS, and YPG. Download FIG S2, PDF file, 1.6 MB. Copyright © 2017 Butts et al. 2017 Butts et al. This content is distributed under the terms of the Creative Commons Attribution 4.0 International license.

### Construction of *Candida albicans* strains with an Erg11p expression differential.

**FIG 2:**
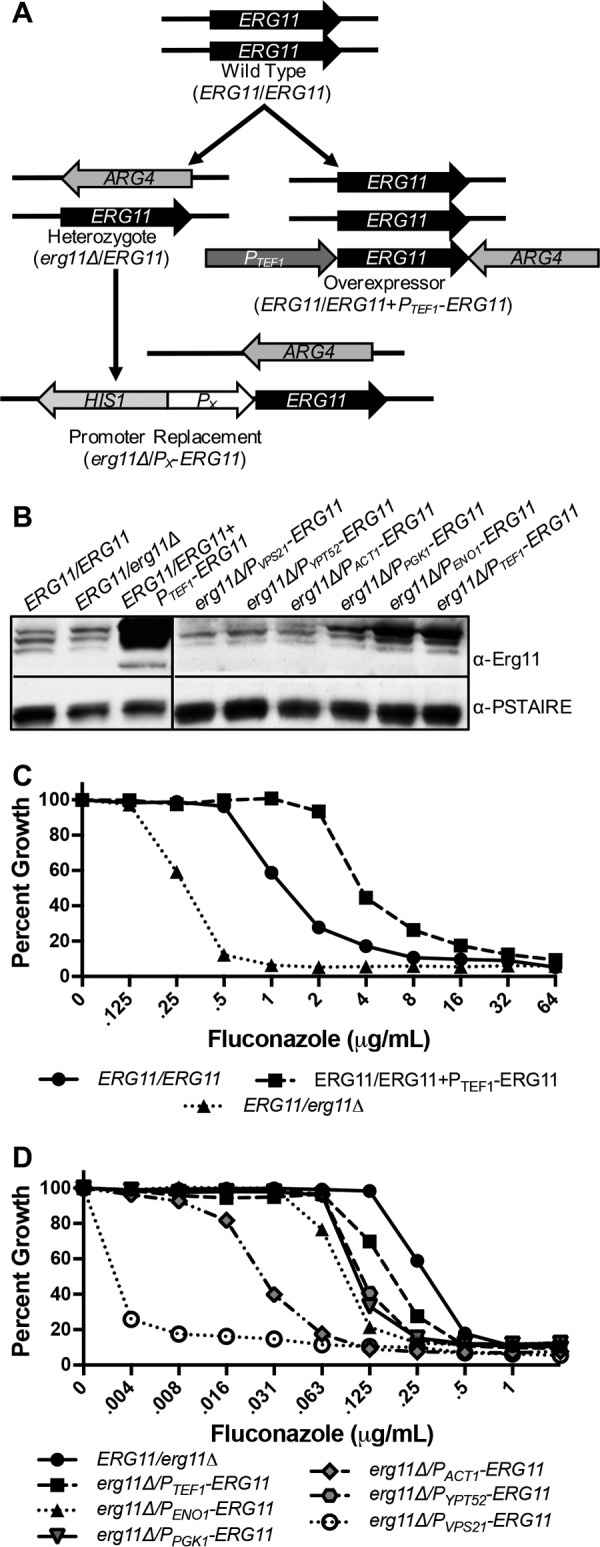
Altering Erg11p expression levels dramatically affects *C. albicans* susceptibility to fluconazole. (A) Schematic of strain construction. (B) *C. albicans* strains were grown into the exponential phase, and whole-cell lysate was prepared and analyzed by Western immunoblotting with anti-Erg11p, as well as anti-PSTAIRE (an internal loading control). (C and D) *C. albicans* strains were grown in YNB at 30°C in the presence of various concentrations of fluconazole. After 48 h, growth was measured as OD_600_ and expressed as a percentage of that of the untreated control. The values presented are the averages of technical triplicates and are representative of two independent experiments.

Lanosterol demethylase (Erg11p), an enzyme involved in the biosynthesis of the membrane lipid ergosterol, is inhibited by the azole antifungals and is the best-characterized drug target described in fungi. We therefore used Erg11p to validate the TAFiS assay and test its reliability to detect on-target inhibitors. An Erg11p overexpression strain was produced by introducing an additional copy of *ERG11* into a wild-type (*ERG11*/*ERG11*) *C. albicans* strain, using an expression vector with the powerful *P*_*TEF1*_ transcriptional promoter. To suppress Erg11p expression, an *ERG11*/*erg11Δ* heterozygous strain was constructed, through replacement of one *ERG11* allele with the *ARG4* selection marker. Since Erg11p is an essential protein (16), it is not possible to construct an *erg11Δ*/*Δ* deletion mutant. Therefore, to further suppress Erg11p expression, the native promoter of the remaining *ERG11* allele in the heterozygous strain was replaced with one of several *C. albicans* promoter sequences (Fig. 2A). This promoter replacement strategy should disrupt the Upc2p-mediated transcriptional activation of *ERG11* that occurs following azole-mediated sterol depletion (17). Such feedback loops directly oppose our goal of maximizing the Erg11p expression differential. In this fashion, a panel of six additional *C. albicans* strains with altered *ERG11* genotypes were generated. Western blot analysis of cell extracts revealed that relative Erg11p expression levels varied by >50-fold among the engineered strains (Fig. 2B).

We next confirmed that changes in Erg11p abundance altered *C. albicans* susceptibility to the on-target inhibitor fluconazole. Dose-response experiments confirmed that fluconazole susceptibility differed by >1,000-fold between the highest- and lowest-Erg11p-expressing strains (Fig. 2C and D). The lowest-Erg11p-expressing strain (*erg11*Δ/*P*_*VPS21*_-*ERG11*) has a pronounced slow-growth phenotype, indicating that the Erg11p level is below some critical threshold required for normal *C. albicans* growth (see Fig. S3 in the supplemental material). Due to its poor growth, the *erg11Δ*/*P_VPS21_-ERG11* strain was not used in subsequent experiments.

10.1128/mSphere.00379-17.4 FIG S3  While the majority of *ERG11* altered expression strains exhibited normal growth, expression of *ERG11* from the *VPS21* promoter substantially altered the growth rate and saturation density. The indicated strains were grown overnight in YPD at 30°C, diluted to an OD of approximately 0.2 in YNB, and inoculated into the wells of a 96-well round-bottom plate. This plate was incubated in the Cyation 5 plate reader at 30°C for 48 h, with optical density being read every hour. The values presented are the averages of technical triplicates (*n =* 3) and are representative of two independent experiments. Download FIG S3, PDF file, 0.2 MB. Copyright © 2017 Butts et al. 2017 Butts et al. This content is distributed under the terms of the Creative Commons Attribution 4.0 International license.

### Chemical-target interactions can be detected as spectroscopic shifts in the FP-tagged expression pools.

**FIG 3:**
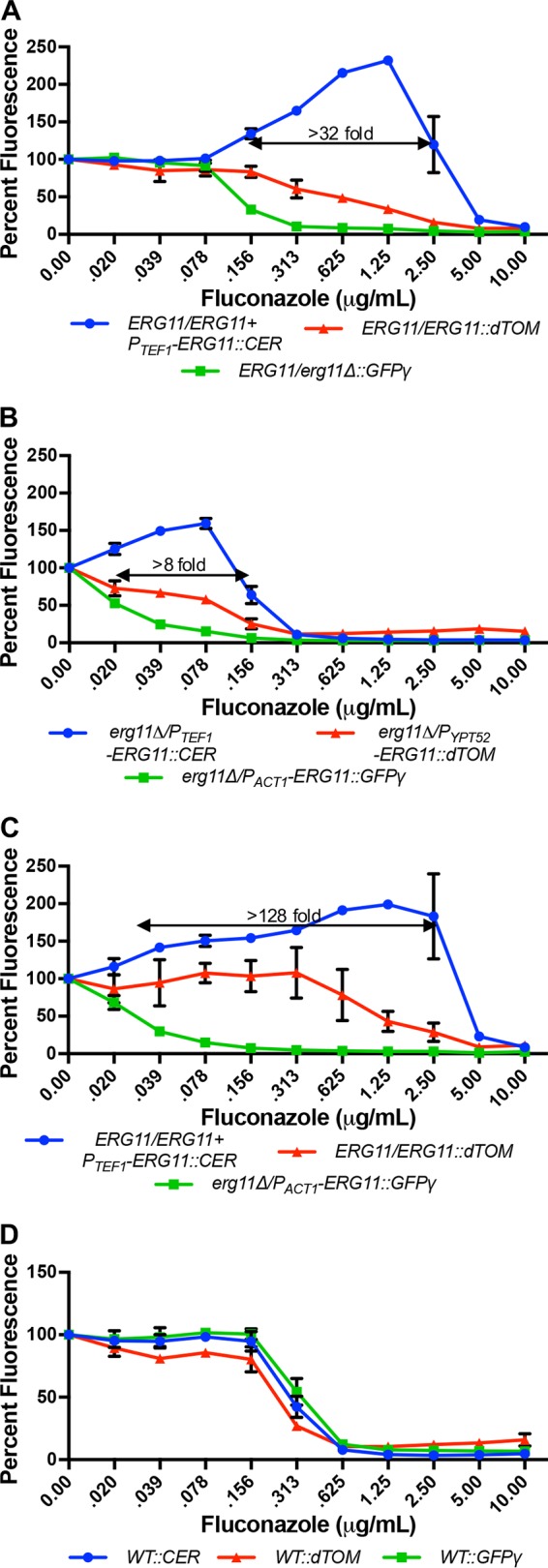
Fluconazole induces a population shift within the FP-tagged Erg11p expression pools over a broad concentration range. Approximately 10^3^ cells of the indicated pool were inoculated into the wells of a 96-well plate in the presence of a range of fluconazole concentrations in YNB. After 48 h at 30°C, fluorescence was read at all three FPs’ wavelengths and expressed as a percentage of fluorescence of the untreated wells. The values presented are the averages and standard deviations of technical triplicates. (A) *C. albicans ERG11*/*ERG11*/*P*_*TEF1*_-*ERG11*::CER (Erg11_Hi_), *ERG11*/*ERG11*::dTOM (Erg11_Med_), and *ERG11*/*erg11*Δ::GFPγ (Erg11_Lo_) strains were mixed in equal proportions to create a gene dosage expression pool. (B) *erg11Δ*/*P_TEF1_-ERG11*::CER (Erg11_Hi_), *erg11Δ*/*P_YPT52_-ERG11*::dTOM (Erg11_Med_), and *erg11Δ*/*P_ACT1_-ERG11*::GFPγ (Erg11_Lo_) promoter replacement strains were combined to create a second expression pool. (C) The *ERG11*/*ERG11*/*P*_*TEF1*_-*ERG11*::CER (Erg11_Hi_) and *ERG11*/*ERG11*::dTOM (Erg11_Med_) strains from the first pool were combined with the *erg11Δ*/*P_ACT1_-ERG11*::GFPγ (Erg11_Lo_) strain from the second pool to create a hybrid expression pool. (D) Three wild-type strains were tagged with CER, dTOM, and GFPγ to create a control pool.

Strains expressing high (Erg11_Hi_), intermediate (Erg11_Med_), and low (Erg11_Lo_) levels of Erg11p were each tagged with an FP expression construct. Two Erg11p expression pools were then created by combining equal proportions of (i) the *ERG11*/*ERG11*/*P_TEF1_-ERG11*::CER (Erg11_Hi_), *ERG11*/*ERG11*::dTOM (Erg11_Med_), and *ERG11*/*erg11Δ*::GFPγ (Erg11_Lo_) gene dosage strains or (ii) The *erg11Δ*/*P_TEF1_-ERG11*::CER (Erg11_Hi_), *erg11Δ*/*P_YPT52_-ERG11*::dTOM (Erg11_Med_), and *erg11Δ*/*P_ACT1_-ERG11*::GFPγ (Erg11_Lo_) promoter replacement strains. These pools were then utilized in coculture experiments to examine how an on-target inhibitor affects the composition of each population. A total of approximately 10^3^ cells from each expression pool, consisting of the three strains in equal proportions, were seeded to the wells of a 96-well plate in YNB medium and grown for 48 h in the presence of various concentrations of fluconazole. Fluorescence intensity was quantified for each tag and compared to the minus-drug (dimethyl sulfoxide [DMSO]) control. As expected, substantive population shifts favoring the Erg11_Hi_ strains were detected in both expression pools over a large range of fluconazole concentrations compared to wells containing DMSO alone (Fig. 3A and B). Similar population shifts, favoring the Erg11_Hi_ strain, were observed using a selection of other azole antifungals, including miconazole and voriconazole (see Fig. S4A and B in the supplemental material). Furthermore, the specific concentration ranges over which the population shifts occurred reflects the relative potency of each azole. When the same dose-response experiments were performed with a pool of three wild-type (*ERG11*/*ERG11*) strains (i.e., with no Erg11p expression differential), tagged with CER, GFPγ, or dTOM, no significant spectral shifts were detected (Fig. 3D). This confirmed that the azole-induced population shifts observed in the Erg11p expression pools are the result of differential Erg11p expression, rather than interference with fluorescent protein signals. However, when similar experiments were performed with terbinafine, a drug that inhibits squalene epoxidase (Erg1p), an enzyme acting upstream of Erg11p (Fig. S4C), or fenpropimorph, which inhibits two enzymes (Erg2p and Erg24p) acting downstream of Erg11p (data not shown), no significant population shifts were detected in the Erg11p expression pool at any concentration. Furthermore, amiodarone and cyclosporine, chemical agents known to synergize with the azole antifungals, did not induce spectral shifts in the tagged Erg11p expression pool (data not shown). Finally, an antifungal drug with an MOA unrelated to ergosterol biosynthesis, caspofungin, equally affected the growth of all strains within the expression pool (Fig. S4D) Collectively these results demonstrate that chemical-induced population shifts within an FP-tagged expression pool indicate a highly specific chemical-target interaction.

10.1128/mSphere.00379-17.5 FIG S4  The azole antifungals induce a population shift within the FP-tagged Erg11p expression pool. The *C. albicans erg11Δ*/*P_TEF1_-ERG11* (Erg11_Hi_), *erg11Δ*/*P_YPT52_-ERG11* (Erg11_Med_), and *erg11Δ*/*P_ACT1_-ERG11* (Erg11_Lo_) *ERG11* promoter replacement strains were tagged with CER, dTOM, and GFPγ, respectively, and combined in equal proportions to create an Erg11p expression pool. Approximately 10^3^ cells of the combined pool were inoculated into YNB medium in the wells of a 96-well plate in the presence of a serial dilution series of (A) miconazole, (B) voriconazole, (C) terbinafine, and (D) caspofungin. After 48 h at 30°C, fluorescence was read at all three FP’s wavelengths and expressed as a percentage of fluorescence of the untreated wells. The values presented are the averages and standard deviations of technical triplicates (*n =* 3) and are representative of two independent experiments. Download FIG S4, PDF file, 0.2 MB. Copyright © 2017 Butts et al. 2017 Butts et al. This content is distributed under the terms of the Creative Commons Attribution 4.0 International license.

### Validation of TAFiS as a chemical screening strategy.

We next determined if TAFiS can provide the basis of a robust HTP chemical screening assay. In order to maximize the Erg11p expression differential and increase the dynamic range of our screen, we created a new hybrid expression pool composed of *ERG11*/*ERG11*/*P_TEF1_-ERG11*::CER (Erg11_Hi_), *ERG11*/*ERG11*::dTOM (Erg11_Med_), and *erg11Δ*/*P_ACT1_-ERG11*::GFPγ (Erg11_Lo_) strains. This pool was then grown in YNB medium in 96-well plates with and without fluconazole. To provide a single metric of comparative strain growth in each well, a relative fitness differential (*R*_*d*_) was calculated as the log_10_ fluorescence intensity ratio of the Erg11_Hi_ and Erg11_Lo_ strains. *Z*′ factors (18) were then calculated from the *R*_*d*_ scores of the Erg11 expression pool grown with and without fluconazole. The *Z*′ factors were 0.86 ± 0.04 with 5 µM fluconazole and 0.57 ± 0.05 with 0.1 µM fluconazole, indicating excellent reproducibility of the assay and confirming its suitability for HTP screening applications.

**FIG 4:**
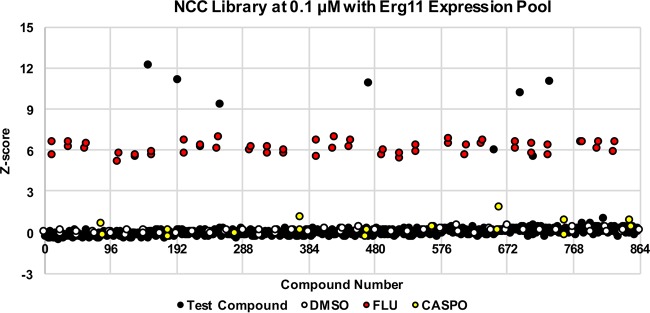
TAFiS can provide a reliable and sensitive HTP screening assay to detect target-specific chemical interactions. An Erg11p expression pool consisting of *ERG11*/*ERG11*/*P*_*TEF1*_-*ERG11*::CER, *ERG11*/*ERG11*::dTOM, and *erg11Δ*/*P_ACT1_-ERG11*::GFPγ cells was used to screen the NCC library in a 96-well format, at a final concentration of 0.1 µM. Based on fluorescence at 48 h, *R*_*d*_ values were calculated for each well and then converted to *Z* scores relative to the collection as a whole, which are plotted above. *Z* scores for the minus-drug control wells (DMSO solvent alone), negative, off-target control wells (0.1 µM caspofungin [CASPO]), and the positive, on-target control wells (0.1 µM fluconazole [FLU]) are also indicated.

Finally, we tested the reliability of a TAFiS-based HTP screen to identify on-target chemical probes. The hybrid Erg11p pool was used to screen the NCC (719 compounds) and Prestwick (1280 compounds) chemical libraries, each of which contains multiple azole antifungals, at final concentrations of either 5 or 0.1 µM. Wells with a total optical density at 600 nm (OD_600_) of <0.1 were discarded from analysis as having populations too small to allow reliable detection of population shifts. *R*_*d*_ scores were calculated for each compound and converted to *Z* scores based on the mean and standard deviation of the collection (Fig. 4). Hits were called based on *Z* scores of >+3 or <−3 in both biological replicates. A complete list of hits with their associated *Z* scores from two separate experiments is provided in Table 1. All nine of the azole antifungals present in the NCC library were identified at one or both of the concentrations screened. Of the 16 azole antifungals found within the Prestwick collection, 14 (87.5%) yielded statistically significant *Z* scores at one or both concentrations. Importantly, only two azole antifungals, butoconazole and itraconazole, failed to yield *Z* scores of >3 at either concentration, likely due to their very high potency. These experiments demonstrate the reliability of TAFiS-based HTP screens to detect on-target chemical probes that are active upon whole cells.

**TABLE 1:** Erg11p expression pool hits^a^

Library	Concn	Compound	*Z* score		
			Trial 1	Trial 2	Mean
NCC	5 µM	Fluconazole	23.8	12.8	18.30
		Bifonazole	7.4	9.7	8.55
	0.1 µM	Oxiconazole	12.1	11.9	12
		Miconazole	11.1	11.5	11.30
		Ketoconazole	10.9	11.4	11.15
		Clotrimazole	10.8	11.2	11
		Econazole	9.2	10.3	9.75
		Voriconazole	10.1	7.5	8.80
		Fluconazole	5.9	6.2	6.05
		Itraconazole	5.5	4.7	5.10

Prestwick	5 µM	Fluconazole	18.7	18.1	18.40
		Enilconazole	15.8	15.6	15.70
		Terconazole	15.2	15.9	15.55
		Bifonazole	11.6	11.6	11.60
		Mupirocin	8.6	9.5	9.05
		Diethylstilbestrol	6.6	7.8	7.20
		Amphotericin B	6.2	6	6.10
		Hexestrol	6.1	4.9	5.50
		Haloprogin	5.2	4.1	4.65
	0.1 µM	Tioconazole	12.3	12.7	12.50
		Sertaconazole	12.4	12.6	12.50
		Oxiconazole	12.4	12.4	12.40
		Miconazole	12	11.9	11.95
		Ketoconazole	11.2	11.7	11.45
		Isoconazole	11.1	11.7	11.40
		Clotrimazole	11.1	11.1	11.10
		Sulconazole	10.9	10.3	10.60
		Econazole	10.4	10.5	10.45
		Fluconazole	7.2	7.8	7.50
		Voriconazole	6.7	6.1	6.40

a Shown are results from compounds identified from the NCC and Prestwick chemical libraries as causing significant population shifts in the Erg11p expression pool.

**FIG 5:**
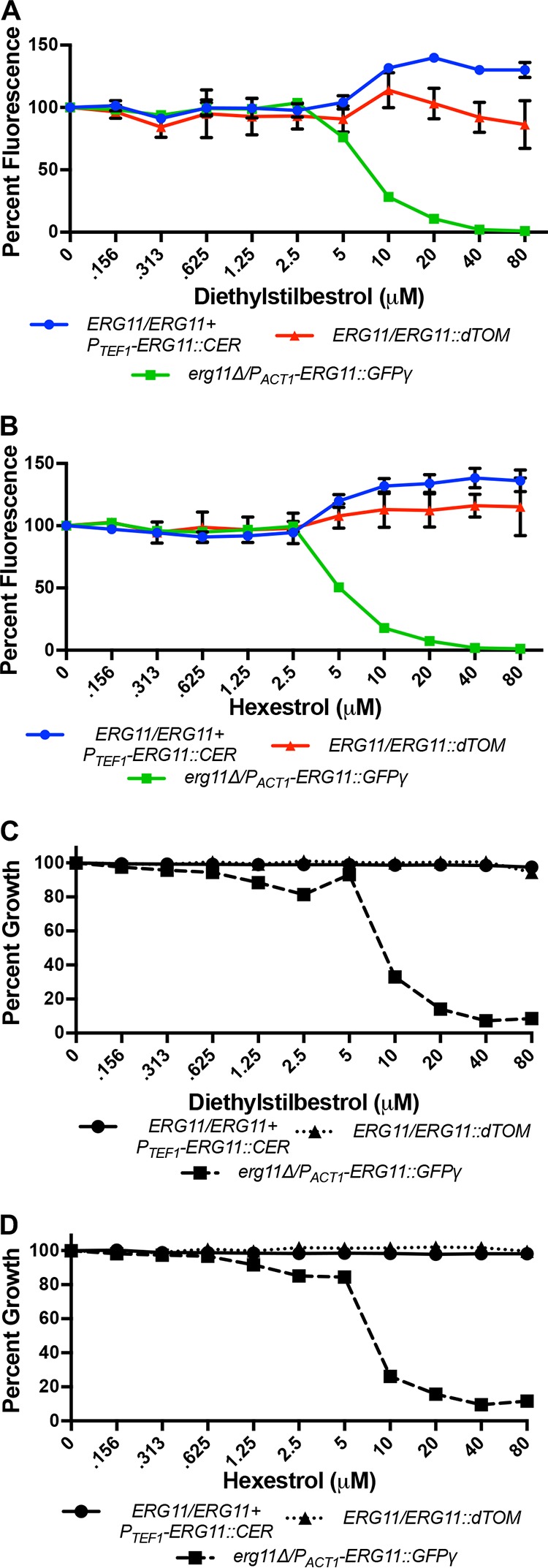
Diethylstilbestrol and hexestrol induce population shifts within the FP-tagged Erg11p expression pools over a broad concentration range. (A and B) *ERG11*/*ERG11*/*P*_*TEF1*_-*ERG11*::CER (Erg11_Hi_), *ERG11*/*ERG11*::dTOM (Erg11_Med_), and *erg11Δ*/*P_ACT1_-ERG11*::GFPγ (Erg11_Lo_) cells were mixed in equal proportions, and approximately 10^3^ cells of this pool were inoculated into the wells of a 96-well plate in the presence of a concentration range of (A) diethylstilbestrol or (B) hexestrol in YNB. After 48 h at 30°C, fluorescence was read at all three FPs’ wavelengths and expressed as a percentage of fluorescence of the untreated wells. The values presented are the averages and standard deviations of technical triplicates. (C and D) Approximately 10^3^ cells of *ERG11*/*ERG11*/*P*_*TEF1*_-*ERG11*::CER (Erg11_Hi_), *ERG11*/*ERG11*::dTOM (Erg11_Med_), or *erg11Δ*/*P_ACT1_-ERG11*::GFPγ (Erg11_Lo_) were inoculated into the wells of a 96-well plate in the presence of a concentration range of (C) diethylstilbestrol or (D) hexestrol in YNB. After 48 h at 30°C, growth was measured as OD_600_ and is expressed as a percentage of that of the untreated control. The values presented are the averages of technical duplicates and are representative of two independent experiments.

In addition to the azole antifungals, five other compounds induced a statistically significant population shift within the Erg11p expression pool. These were only detected at the higher screening concentration (5 µM) and with less significant *Z* scores than the azoles. Nonetheless, among these were amphotericin B, an antifungal that directly binds ergosterol in the fungal plasma membrane (Table 1), and a second antifungal, haloprogin, of unknown mechanism. Two closely related synthetic nonsteroidal estrogens, diethylstilbestrol and hexestrol, were also identified and selected for follow-up analysis. Dose-response assays with the Erg11p expression pool confirmed that both compounds induced a spectral shift over an 8-fold concentration range (Fig. 5A and B). In contrast, neither drug induced a shift in the tagged wild-type control pool, confirming that the observed shifts depend upon differential Erg11p expression levels and are not an indirect consequence of spectral interference.

Dose-response experiments with individual strains revealed that both compounds were growth inhibitory toward the low-expression strain, but neither was sufficient to inhibit the growth of wild-type *C. albicans* at concentrations up to 50 µM (Fig. 5C and D). To determine if either diethylstilbestrol or hexestrol is a bona fide inhibitor of ergosterol biosynthesis, sterol profiles of wild-type cells treated with either compound were analyzed. Treatment with either diethylstilbestrol or hexestrol resulted in a decrease in ergosterol content and a substantial buildup of lanosterol (Table 2), a profile consistent with partial Erg11p inhibition, indicating they both engage the intended target enzyme.

**TABLE 2:** Membrane sterol profiles^a^

Sterol	% of total sterols in:									
	DMSO		5 µM DSB		50 µM DSB		5 µM HEX		50 µM HEX	
	Mean	SD	Mean	SD	Mean	SD	Mean	SD	Mean	SD
Ergosta-5,8,22,24(28)-tetraenol	2.2	1.3	2.3	1.4						
Zymosterol	8.2	0.1	4.4	0.1	2.9	0.2	6.6	0.9	4.4	0.8
Ergosterol	64.1	0.8	65.1	2.4	59.8	3.5	65.5	1.8	55.4	4.7
Ergosta-7,22-dienol	1.0	0.2	0.8	0.1						
Ergosta-5,7,22,24(28)-tetraenol	1.0	0.2	1.1	0.3	1.1	0.3	1.2	0.4	1.7	1.3
Fecosterol	2.6	0.2	1.2	0.0			1.7	0.1		
14-Methyl fecosterol			0.5	0.0	1.7	0.3			2.2	0.2
Ergosta-5,7-dienol	4.8	0.6	3.3	0.3	2.2	0.4	4.4	0.2	2.9	0.6
Episterol	4.6	0.5	2.5	0.1	1.6	0.1	3.2	0.7	2.1	0.2
Lanosterol	6.9	0.6	15.2	1.8	28.4	2.9	13.7	3.3	28.4	5.9
4,4-Dimethyl cholesta-8,24-dienol	4.7	0.4	3.8	0.6	2.3	0.4	3.7	1.7	3.0	0.7

a Treatment of wild-type *C. albicans* with either diethylstilbestrol (DSB) or hexestrol (HEX) results in altered sterol composition consistent with Erg11p inhibition.

### Validation of TAFiS with dihydrofolate reductase.

**FIG 6:**
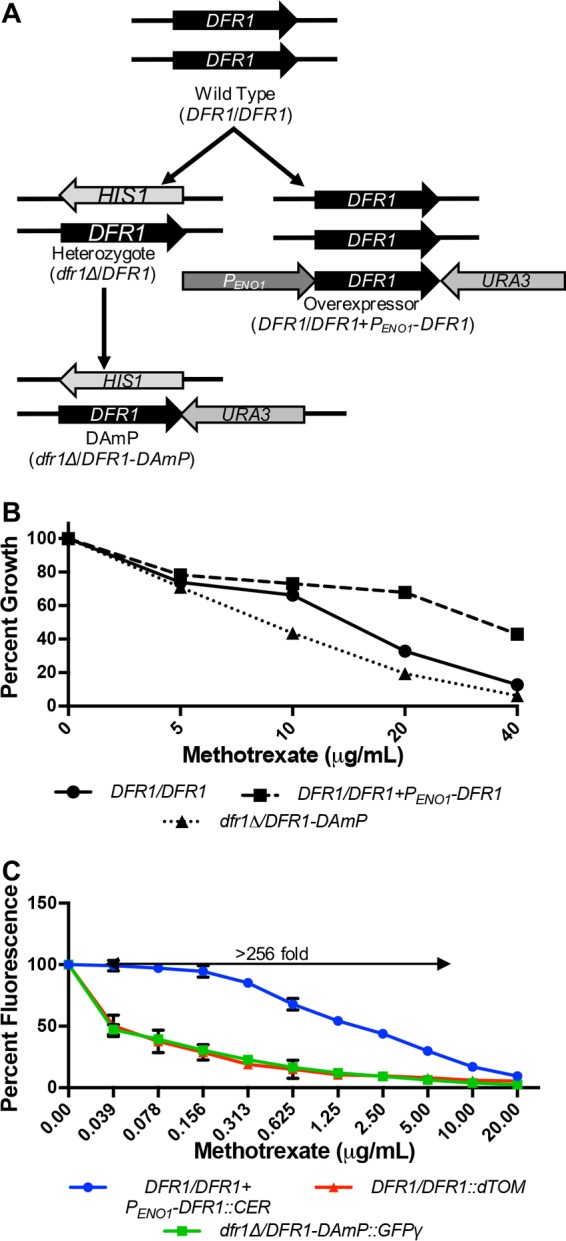
Modulation of Dfr1p expression affects *C. albicans* susceptibility to methotrexate. (A) Schematic of strain construction. (B) *C. albicans* strains were grown in YNB at 30°C in the presence of various concentrations of methotrexate. After 48 h, growth was measured via OD_600_ and expressed as a percentage of that measured in the untreated control. The values presented are the averages of technical triplicates. (C) *C. albicans DFR1*/*DFR1*/*P*_*ENO1*_-*DFR1*::CER (Dfr1_Hi_), *DFR1*/*DFR1*::dTOM (Dfr1_Med_), and *dfr1*Δ/*DFR-*DAmP::GFPγ (Dfr1_Lo_) strains were combined to create an expression pool. Approximately 1 × 10^3^ cells of the pool were inoculated into the wells of a 96-well plate in the presence of a range of methotrexate concentrations in YNB. After 48 h at 30°C, fluorescence was read at all three FPs’ wavelengths and expressed as a percentage of fluorescence of the untreated wells. The values presented are the averages and standard deviations of technical triplicates.

To further validate TAFiS as a viable chemical screening strategy, we applied it to dihydrofolate reductase (Dfr1p), another historically important target for antimicrobial drug development (19–21). A *C. albicans* Dfr1p expression pool was produced that included an overexpression strain (Dfr1_Hi_ [*DFR1*/*DFR1*/*P*_*ENO1*_-*DFR1*]), a heterozygous strain (Dfr1_Med_ [*DFR1*/*dfr1Δ*]), and a knockdown strain (Dfr1_Lo_ [*dfr1*Δ/*DFR1-*DAmP]). The knockdown strain was produced using a technique known as decreased abundance by mRNA perturbation (DAmP) (22), to destabilize the mRNA transcript (Fig. 6A). Quantitative reverse transcription-PCR (qRT-PCR) confirmed a 30-fold differential in *DFR1* mRNA abundance between Dfr1_Hi_ and Dfr1_Lo_ strains (data not shown). Significant population shifts within the Dfr1p expression pool were detected over a >256-fold concentration range of the known Dfr1p inhibitor methotrexate (MTX) (Fig. 6C). Treatment of the tagged Dfr1p pool with 5 µM methotrexate yielded a *Z*′ factor of 0.63 ± 0.05 in 96-well plate-based assays, again supporting the excellent quality of the assay. Finally, the tagged Dfr1p expression pool was used to screen the same chemical libraries described above at a final concentration of 5 µM. Only methotrexate produced a positive *Z* score of ≥3. (Note that amethopterin, also identified as a hit, is the same chemical entity.) Unexpectedly, 21 compounds induced a statistically significant inverse population shift (*Z* scores of ≤−3), indicating that the Dfr1_Hi_ strain was more sensitive than the Dfr1_Lo_ strain (Table 3).

**TABLE 3:** Dfr1p expression pool hits^a^

Library	Concn	Compound	*Z* score		
			Trial 1	Trial 2	Mean
NCC	5 µM	Methotrexate	4.0	4.4	4.2
		Voriconazole	−3.7	−3.0	−3.4
		Miconazole	−3.5	−3.7	−3.6
		Triclabendazole	−3.3	−4.3	−3.8
		Disulfiram	−3.8	−4.9	−4.3
		Mupirocin	−4.2	−5.2	−4.7
		Fluconazole	−5.2	−4.4	−4.8
		Oligomycin A	−5.2	−7.3	−6.3
		Fluvastatin	−7.0	−6.2	−6.6
		Cerivastatin	−7.3	−7.8	−7.6
		Hexachlorophene	−11.0	−5.0	−8.0

Prestwick	5 µM	Amethopterin	5.9	6.8	6.3
		Methotrexate	5.5	6.8	6.1
		Pyrvinium	−3.5	−4.5	−4.0
		Atorvastatin	−4.3	−4.0	−4.1
		Pemetrexed	−3.4	−6.4	−4.9
		Benzethonium	−3.2	−8.0	−5.6
		Haloprogin	−5.7	−6.0	−5.8
		Disulfiram	−5.5	−6.4	−5.9
		Chloroxine	−4.7	−7.3	−6.0
		Monensin	−5.8	−9.4	−7.6
		Merbromin	−7.4	−10.9	−9.1

a Shown are results from compounds from the NCC and Prestwick chemical libraries identified as causing significant population shifts in the Dfr1p expression pool.

### Adapting TAFiS to a 384-well format.

In order to increase throughput and efficiency, we examined the performance of the TAFiS assay in 384-well plates using the tagged Erg11p expression pool. Using volumes as low as 20 µl, the tagged *C. albicans* strains demonstrated excellent signal/background ratios versus the untagged controls (data not shown). Furthermore, the Erg11p pool yielded *Z*′ factors of 0.78 ± 0.03 and 0.70 ± 0.05 when treated with 5 and 0.1 µM fluconazole, respectively, indicating excellent performance in this higher-density format. In conclusion, we have demonstrated that TAFiS-based HTP assays can provide a reliable and highly sensitive approach to efficiently identify physiologically active and target-specific chemical probes.

## DISCUSSION

The enormous costs and prolonged timelines associated with developing a new pharmacotherapy underscore the desperate need to increase the efficiency of both preclinical and clinical phases of drug discovery and development. Herein we describe a new type of TB-WCS that can greatly expedite this process through the selection of physiologically active and target-specific hits at the earliest possible stage, the primary screen. TB-WCS assays have several major advantages over conventional target-based and cell-based screening strategies and to a large extent combine the benefits of both into a single screening assay. First, chemical probes are selected that functionally interact with a defined molecular target, providing invaluable insight into each hit’s MOA from the outset. Second, the selected target is presented within its native environment, and thus only physiologically active small molecules that can access and engage the target in its cellular context are recognized. Third, as the measured outcome is comparative growth of strains expressing differing levels of target protein, TB-WCS does not require purification of the target protein itself or a specific and HTP-compatible biochemical assay of its activity in order to identify relevant chemical-target interactions. Indeed, this approach can readily be applied to targets that are not amenable to biochemical analysis, such as noncatalytic proteins. Fourth, in addition to identifying compounds that directly engage the selected target protein, TB-WCS assays have the potential to identify compounds that indirectly interact with the selected target protein. For instance, compounds that act upon compensatory or redundant pathways that are required for survival when the primary target protein is limiting may be expected to preferentially inhibit the growth of the knockdown strain. Compounds that act by such indirect interactions are potentially of great value as they may uncover new functional interactions between distinct cellular pathways or enhance the efficacy of on-target drugs and thus provide a basis for combination therapy. As such, TB-WCS approaches have the potential to yield a diverse set of physiologically relevant chemical probes that functionally interact with the selected target protein from a single primary screen. Finally, compounds that lack specificity (e.g., that engage multiple targets) or which are generally toxic to cells are unlikely to create the target-dependent fitness differential upon which these approaches depend and are thus eliminated at the primary screening stage. Despite enormous potential advantages, TB-WCS approaches are underutilized as a drug discovery strategy. While several important studies in the pathogenic prokaryotes *Staphylococcus aureus* and *Mycobacterium tuberculosis* have collectively established the validity of TB-WCS assays to discover on-target chemical probes (6, 23, 24), each has been restricted by several technical issues that have limited efficiency, sensitivity, and/or throughput of the screens. The approach described here builds upon previous efforts but also incorporates several technological advances that improve sensitivity and efficiency. First, nearly all TB-WCS assays that have been described to date compared only the growth of a knockdown strain, with reduced target expression, to a reference strain expressing normal levels of the target protein. In our approach, the effects of each test compound on both target knockdown and overexpression strains are simultaneously compared to a reference strain. This should enhance both the dynamic range and sensitivity of the screening assay to detect functionally interacting chemicals. Second, previous studies invariably used two-plate assays that compare the growth of the reference and knockdown strains on separate culture plates. With TAFiS, the overexpression, reference, and knockdown strains are cocultured, and thus the relative growth of each strain is compared in a single well to improve screening efficiency. Furthermore, the competitive fitness basis of the TAFiS assay enhances the sensitivity of this approach compared to previous two-plate assays, as demonstrated by the identification of two previously uncharacterized Erg11p inhibitors at sub-growth-inhibitory concentrations. Third, previous TB-WCS assays were based on measurements such as colony size or zones of growth inhibition. These outcomes are poorly suited to numerical measurement, automated data collection, and statistical analysis. Through the incorporation of fluorescent protein tags, TAFiS facilitates straightforward, quantitative measurements of the relative growth of each strain, providing parameters that are readily amenable to statistical analysis. This in turn simplifies the selection, ranking, and prioritization of hits according to the magnitude of the observed population shift and by inference the strength of the compound-target interaction.

In selecting a well-characterized target protein (Erg11p) to validate the TAFiS methodology, a large number of on-target inhibitors were present within the available chemical libraries. However, due to their extreme potency, several of the highly optimized azole antifungals apparently overwhelmed all strains within the Erg11p expression pool, and thus no differential susceptibility/population shift was observed at the standard screening concentration of 5 µM. Accordingly, repeating the screen at a lower concentration of 0.1 µM actually identified more of the on-target azoles than at the higher concentration. We anticipate that the occurrence of such extremely potent on-target hits is unlikely when applying TAFiS to novel target proteins or when screening unoptimized chemical libraries. Thus, we expect standard 5 to 10 µM compound concentrations to be appropriate for most TAFiS-based primary screens. Additionally, these libraries both contain a number of highly growth-inhibitory compounds of a variety of mechanisms, which is unlikely to pose significant complications when screening libraries that have not been enriched for bioactive compounds as these collections have been. We also chose to screen two chemical libraries with significant overlap. Since hits were defined relative to the collection, there were slightly different thresholds of significance for each library that probably account for the few discrepancies between the hit lists for each.

As stated above, the potential exists for TAFiS to identify chemical probes that directly engage the target protein as well as those that indirectly interact with the target protein’s function: for example, by acting upon redundant or compensatory pathways. However, our studies with Erg11p and Dfr1p indicate that TAFiS is exceptionally reliable at identifying on-target chemical probes, with few off-target hits identified as causing positive population shifts (i.e., favoring the T_Hi_ strain) in either screen. This is particularly reassuring, given that suppression of Erg11p activity is expected to alter membrane sterol composition and consequently plasma membrane permeability (25). In such a situation, differential permeability of the strains within the Erg11p expression pool might be expected to elevate the number of off-target compounds identified. However, our results suggest this was a not major complication. Intriguingly, several compounds were identified that resulted in negative *Z* scores with the Dfr1p expression pool, indicating that the Dfr1_Hi_ strain is more susceptible to these agents than the Dfr1_Lo_ strain. The exact meaning of these interactions is currently unclear; however, it likely reflects the important role of folate in a myriad of metabolic processes. Nonetheless, these preliminary studies collectively support that TAFiS can provide an extremely reliable and efficient means to identify physiologically active and target-selective chemical probes.

One potential concern with TAFiS is that small molecules may interact with or selectively quench the fluorescent protein tags or that fluorescence of a subset of compounds may skew the FP signal intensity, resulting in false positives. However, this was not a major problem in our chemical screens with Erg11p or Dfr1p, which used cerulean, GFPγ, and dTomato to tag the individual strains. More recently, we have identified phi yellow fluorescent protein (φYFP) (13) as an exceptionally bright FP tag under our standard TAFiS conditions. This will now enable the selection of closely related fluorescent proteins (e.g., CER, GFPγ, and φYFP), which should ensure that selective fluorescent protein interactions are a rare occurrence. Nonetheless, such false positives can be rapidly eliminated using a simple counterscreen—testing the effect of each initial hit on an FP-tagged control pool of strains with no target protein expression differential. Chemicals that induce spectral shifts in this control pool interfere with the fluorescent signal or detection and should be eliminated from further analysis. Finally, the field of fluorescent protein engineering (26, 27), as well as the instruments used for their detection, is rapidly evolving. We anticipate that as brighter FPs with more defined spectral profiles become available, the potential to multiplex TAFiS (i.e., to simultaneously screen multiple expression pools each representing a different target protein, within a single well) will grow. Such multiplexing may for example facilitate the simultaneous screening against all components of a pathway and will yield further cost and time savings.

Moving forward, when applying TAFiS to additional systems, it is crucial to remember that there are a variety of both target- and compound-specific factors that may influence both the magnitude and directionality of the observed population shifts. Target-specific factors may include the magnitude of the target expression differential, the consequences of hypo- and hyperactivity of the target protein under assay conditions, and the subunit composition of the target. Maximizing the target protein expression differential between the T_Hi_ and T_Lo_ expression strains should theoretically increase the range of concentrations over which an on-target probe differentially impacts their fitness and thus the dynamic range of the screen. In the case of an essential gene, this is limited by the lower threshold of target protein activity that is sufficient to sustain cell viability. However, in the case of a nonessential protein, a target gene deletion strain can be incorporated into the expression pool. Accordingly, the population shifts expected to occur in response to a directly interacting chemical probe would be different, in that a target deletion strain should be insensitive to on-target modulators. Chemical-specific factors that may influence outcomes in the TAFiS assay include the strength of the chemical-target interaction, the precise mode of action (e.g., inhibition versus activation), and the specificity of target engagement. Finally, TAFiS is theoretically applicable to any microbial species that is culturable and genetically tractable. We anticipate the development of closely related methodologies in a variety of other microbes, including bacterial, protozoan, and fungal pathogens. Due to the ease of recombinant protein expression in the yeast *Saccharomyces cerevisiae*, adaptation of TAFiS to this system could facilitate chemical screens using targets derived from nonculturable microbes, species not amenable to genetic manipulation, or even disease-relevant human proteins.

## MATERIALS AND METHODS.

### Growth conditions.

*C. albicans* was routinely grown on yeast extract-peptone-dextrose (YPD) agar plates at 30°C, supplemented with 50 μg/ml uridine for *ura3*^−^ strains. Selection of *C. albicans* transformants was carried out on minimal YNB medium (6.75 g/liter yeast nitrogen base without amino acids, 2% dextrose, 2% Bacto agar) supplemented with the appropriate auxotrophic requirements described for *S. cerevisiae* (28) or with 50 μg/ml uridine.

### Plasmid construction.

Plasmid pLUX (29) was kindly provided by William Fonzi (Georgetown University), pMG2254 (8) and pMG1648 (30) were acquired from the Fungal Genetics Stock Center, pENO1-dTom-NATr (9) was acquired from Addgene, pGateway-TagBFP (10) was acquired from Evrogen, pFA-GFPγ (11) was kindly provided by James Konopka (Stony Brook University), and pGEMHIS1, pDDB57, and pRSARG4ΔSpeI (31, 32), were kindly provided by Aaron Mitchell (Carnegie Mellon University). The *C. albicans* expression vectors pKE1 (*ACT1pr*) (33) and pKE3 (*ENO1pr*) (34) have been previously described. A detailed description of all other plasmid vectors constructed as part of these studies is provided in Text S1 in the supplemental material. All oligonucleotides used in this study are listed in Table S1 in the supplemental material.

10.1128/mSphere.00379-17.1 TEXT S1  Supplemental materials and methods. Plasmid and disruption cassette construction. Download TEXT S1, DOCX file, 0.1 MB. Copyright © 2017 Butts et al. 2017 Butts et al. This content is distributed under the terms of the Creative Commons Attribution 4.0 International license.

10.1128/mSphere.00379-17.7 TABLE S1  Oligonucleotides used in this study. *, engineered restriction enzyme sites are underlined. Download TABLE S1, DOCX file, 0.1 MB. Copyright © 2017 Butts et al. 2017 Butts et al. This content is distributed under the terms of the Creative Commons Attribution 4.0 International license.

10.1128/mSphere.00379-17.8 TABLE S2  Coding sequence for synthetic *Candida albicans* optimized FP coding sequences. Download TABLE S2, DOCX file, 0.2 MB. Copyright © 2017 Butts et al. 2017 Butts et al. This content is distributed under the terms of the Creative Commons Attribution 4.0 International license.

### *C. albicans* strain construction.

BWP17 (32) and CAI4 (35), were kindly provided by Aaron Mitchell (Carnegie Mellon University) and William Fonzi (Georgetown University), respectively. *C. albicans* was transformed with DNA constructs using the lithium acetate procedure (36). Gene deletion strains were constructed using the PCR-based approach described by Wilson et al. (32). All pKE3- and pKE4-based vectors (*URA3* selection marker), including pKE3-DFR1 and the pKE4-based FP expression constructs, were cut with NheI prior to transformation of *ura3*^−^ recipient *C. albicans* strains to target integration at and fully restore the *URA3-IRO1* locus. All pAR8-based vectors (*ARG4* selection marker), including the pAR8-ERG11 and the pAR8-based FP expression constructs, were cut with ClaI prior to transformation of *arg4*^−^ recipient *C. albicans* strains, to target integration at and restoration of the *ARG4* locus. A detailed description of how each gene deletion, target overexpression, promoter replacement, and FP-tagged *C. albicans* strain was constructed is provided in Text S1.

### Comparison of FP tag brightness.

Several *C. albicans* transformants expressing each FP tag were grown overnight in YPD at 30°C, diluted to approximately 5 × 10^3^ cells/ml in YNB medium, and 200 μl of each cell suspension was transferred into the wells of a round-bottom 96-well plate. Following incubation at 30°C for 48 h, fluorescence intensity at the appropriate wavelengths and OD_600_ were measured using a Cytation 5 plate reader (Bio-Tek Instruments, Inc.). The excitation/emission wavelengths used for tagBFP, CER, GFPγ, YFP, φYFP, ZsYellow, dTOM, mCherry, and mPlum were 402/457, 433/475, 488/507, 510/531, 529/550, 533/558, 554/581, 587/612, and 590/649, respectively, using a 9-nm bandwidth for both excitation and emission wavelengths. Fluorescence intensity was then normalized to growth (OD_600_) and expressed relative to background fluorescence, as measured in an isogenic, vector-alone control strain. For the purpose of pooled experiments involving both φYFP- and dTOM-tagged strains, suboptimal wavelengths of 560/590 with 9-nm bandwidth were used for dTOM to minimize spectral overlap.

### Antifungal susceptibility testing.

Stock solutions of fluconazole, miconazole, voriconazole, terbinafine, methotrexate, and caspofungin (Sigma-Aldrich) were prepared at 10 mg/ml in DMSO and diluted as needed in the same solvent. Each *C. albicans* strain was grown overnight in YPD at 30°C and diluted to 1 × 10^4^ cells/ml in YNB medium, and 100 μl of each cell suspension was transferred to the wells of a round-bottom 96-well plate. An additional 100 μl of YNB medium containing 2× the final desired concentration of each drug was then added to each well. The final concentration of DMSO was 0.5% for all treatments. Plates were then incubated at 30°C before growth was quantified after 24 and 48 h by measuring OD_600_ using a Cytation 5 plate reader (Bio-Tek Instruments, Inc.). The growth of each strain at each drug concentration was then expressed relative to the minus-drug (DMSO-alone) control.

The multistrain competition-based experiments were performed as described above, except the individual FP-tagged strains were diluted to 1 × 10^4^ cells/ml, mixed 1:1:1 to create the three-member expression pools, before they were dispensed into the 96-well plates. Following incubation at 30°C for 48 h, OD_600_ and fluorescence intensity were measured as described above. The relative growth of each strain in the presence of drug was then expressed as a percentage of the same strain’s growth in the minus-drug (DMSO-alone) control coculture, as measured by the fluorescence intensity of the corresponding FP tag.

### Stress phenotypes.

Each *C. albicans* strain was grown overnight in YPD at 30°C, the cell density was adjusted to 10^8^ cells/ml in sterile water, and serial 1:10 dilutions were performed in a 96-well plate. Cell suspensions were then applied to agar plate surfaces using a sterile multipronged applicator. Resistance to temperature stress was determined on YPD agar at 37 and 42°C, resistance to osmotic stress was determined on YPD agar supplemented with 1 M sorbitol, and resistance to ionic stress was determined on YPD agar plus 1 M NaCl. Sensitivity to metal ion stress was also tested on YPD agar supplemented with either 50 μM CuCl_2_, 50 μM ZnCl_2_, or 10 mM MnCl_2_. Sensitivity to cell wall and membrane stress was compared on YPD agar supplemented with either 5 mM caffeine, 100 μg/ml Congo red, or 0.05% SDS. The ability to utilize nonfermentable carbon sources was also compared on YPG agar (YPD agar with 3% glycerol in place of dextrose).

### Western immunoblot analysis.

Each *C. albicans* strain was grown overnight in YPD at 30°C and then subcultured to an OD_600_ of 0.2 in 10 ml of fresh YPD and grown for an additional 6 h at 30°C and collected via centrifugation. Total protein extracts were prepared by resuspending cells in 200 μl lysis buffer (50 mM Tris-HCl [pH 7.5], 150 mM NaCl, 1 mM EDTA, 1% Triton X-100) with added protease inhibitor cocktail (Roche) and 0.5-mm-diameter glass beads. Cells were lysed by 10 cycles of 20-s bursts with a bead beater followed by 60 s on ice. The total protein concentration was determined using a Bradford assay (Thermo Scientific) dye concentrate according to the manufacturer’s instructions. Fifty micrograms of each protein lysate was fractionated on a 10% SDS-PAGE Mini-Protean TGX gel (Bio-Rad), transferred to a nitrocellulose membrane, and blocked with 5% nonfat milk in 50 mM Tris (pH 7.5), 150 mM NaCl, and 0.05% Tween 20 (TBST). The membranes were then probed with an anti-Erg11 antibody (generously provided by Steve Kelly, Swansea University) (37) followed by anti-rabbit horseradish peroxidase (HRP)-conjugated secondary antibody (Bio-Rad) and with anti-PSTAIRE (Abcam, Inc.) antibody, which binds to that consensus sequence, followed by anti-mouse HRP-conjugated secondary antibody (Bio-Rad) as an internal loading control. HRP conjugates were detected with the Clarity ECL Western blotting detection system (Bio-Rad). Blots were imaged using a G:Box Chemi XT-4 (Syngene) and analyzed using Image Studio Lite (Li-Cor).

### RNA isolation and qRT-PCR.

Each *C. albicans* strain was grown overnight in YPD at 30°C and then subcultured to an OD_600_ of 0.2 and incubated at 30°C with shaking for 6 h. Cells were pelleted by centrifugation before total cellular RNA was extracted by the hot phenol method (38). cDNA was synthesized from total RNA using the Verso cDNA synthesis kit (Thermo Scientific), in accordance with the manufacturer’s instructions. Synthesized cDNA was used for the amplification of *ACT1* and the gene of interest by PCR, using SYBR green PCR master mix, according to the manufacturer’s instructions. Gene-specific primers were designed using the PrimerQuest Tool from IDT, synthesized by Integrated DNA Technologies, Inc., and are listed in Table S1. The threshold cycle (2^−ΔΔ*CT*^) method was used to calculate changes in gene expression among the strains (39). All experiments included both biological and technical replicates in triplicate.

### Chemical libraries.

The NIH Clinical Collection (NCC) library of 719 small molecules was provided by the NIH Small Molecule Repository. The Prestwick library of 1,280 small molecules was purchased from Prestwick Chemical. All library compounds were supplied at 10 mM in DMSO in a 96-well plate format. These were diluted further in DMSO to final concentrations of 1 or 0.02 mM, and 1-µl volumes were dispensed into round-bottom 96-well plates that were used for the chemical screens.

### Chemical screening.

The FP-tagged *C. albicans* strains expressing low, intermediate, and high levels of target protein were grown overnight in YPD at 30°C, diluted to approximately 5 × 10^3^ cells/ml in YNB medium, and mixed 1:1:1 to create the three-member expression pools. One hundred ninety-nine microliters of the mixed cell suspension (~10^3^ cells) was then added to each well of each library plate, resulting in final compound concentrations of 5 or 0.1 μM. After 24 and 48 h of incubation at 30°C, the OD_600_ and fluorescence at the appropriate wavelengths were measured at as described above. The relative fitness differential (*R*_*d*_) was then calculated for each well as the log_10_ T_Hi_/T_Lo_ fluorescence and converted to Z scores using the average and standard deviation of the *R*_*d*_ score for the whole collection. Hits were called based on a *Z* score of >+3 or <−3 in two independent replicates. Wells with little detectable *C. albicans* growth (OD_600_ of <0.1) were discarded from analysis.

### Sterol extraction and analysis.

Twenty-five milliliters of YNB containing 1% DMSO and the indicated concentration of diethylstilbestrol or hexestrol was inoculated with SC5314 at a concentration of 1 × 10^3^ cells/ml. Cultures were incubated at 37°C for 18 h. Nonsaponifiable lipids were extracted using alcoholic KOH. Samples were dried in a vacuum centrifuge (Heto) and were derivatized by the addition of 100 µl 90% *N*,*O*-bis(trimethylsilyl)trifluoroacetamide (BSTFA)–10% trimethylsilyl (TMS [Sigma]) with 200 µl anhydrous pyridine (Sigma) and heating for 2 h at 80°C. TMS-derivatized sterols were analyzed and identified by gas chromatography-mass spectrometry (GC-MS) (Thermo 1300 gas chromatograph coupled to a Thermo ISQ mass spectrometer; Thermo Scientific) with reference to retention times and fragmentation spectra for known standards. GC-MS data files were analyzed using Xcalibur software (Thermo Scientific) to determine sterol profiles for all isolates and for integrated peak areas (40).

10.1128/mSphere.00379-17.6 FIG S5  TAFiS can provide a reliable and sensitive HTP screening assay to detect target-specific chemical interactions. (A) A tagged Erg11p expression pool consisting of equal fractions of *ERG11*/*ERG11*/*P*_*TEF1*_-*ERG11*::CER, *ERG11*/*ERG11*::dTOM, and *erg11Δ*/*P_ACT1_-ERG11*::GFPγ cells and (B) a tagged Dfr1 expression pool consisting of equal fractions of *DFR1*/*DFR1 P*_*ENO1*_-*DFR1*::CER, *DFR1*/*DFR1*::dTOM, and *dfr1*Δ/*DFR*-DAmP::GFPγ cells were used to screen the NCC library of 719 compounds in 96-well format, at a final compound concentration of 5 µM. After 48 h of growth, the fluorescence of each tag was quantified in each well. A relative fitness differential (*R*_*d*_) was then calculated for each well as the log_10_ T_Hi_/T_Lo_ fluorescence ratio. *R*_*d*_ scores for each well were then expressed as *Z* scores based on the average and standard deviation of *R*_*d*_ scores across the entire collection, to identify wells in which a statistically significant population shift occurred. *Z* scores for the minus-drug control wells (DMSO solvent alone), positive-control wells (5 µM fluconazole and methotrexate, respectively), and the growth controls (5 µM caspofungin) are also indicated. Download FIG S5, PDF file, 0.4 MB. Copyright © 2017 Butts et al. 2017 Butts et al. This content is distributed under the terms of the Creative Commons Attribution 4.0 International license.
