# Supplementary figures and images for: Target Abundance-Based Fitness Screening (TAFiS) Facilitates Rapid Identification of Target-Specific and Physiologically Active Chemical Probes
Source: mSphere. 2017 Oct 4;2(5):e00379-17. doi: 10.1128/mSphere.00379-17 (PMC5628291; doi:10.1128/mSphere.00379-17)

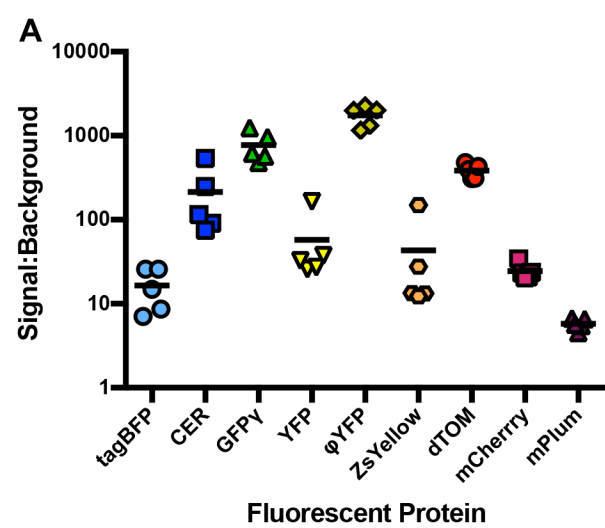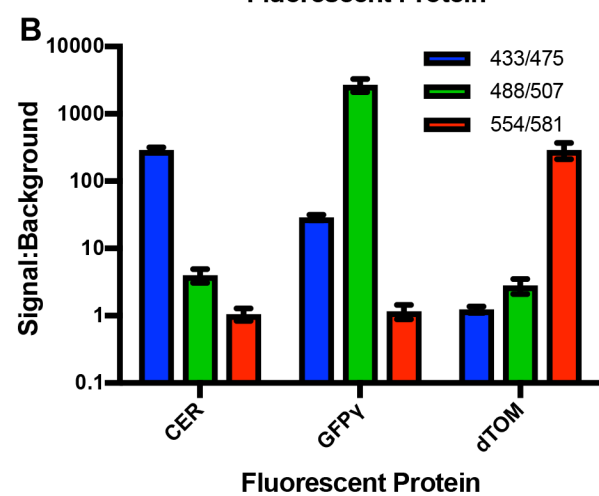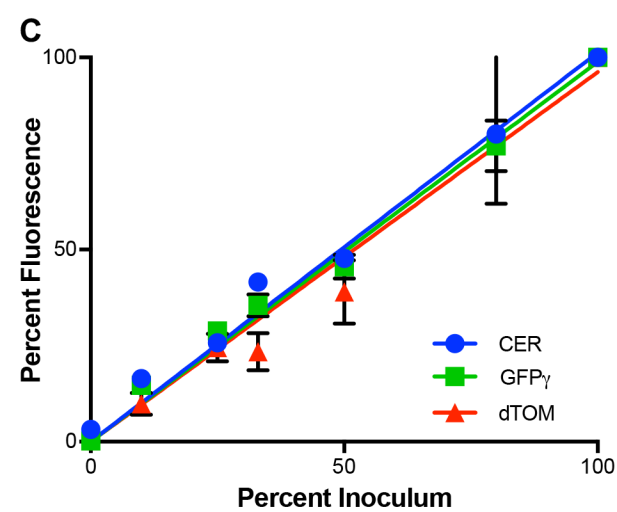

Supplement: FIG S1 [file sph005172377sf1.pdf]

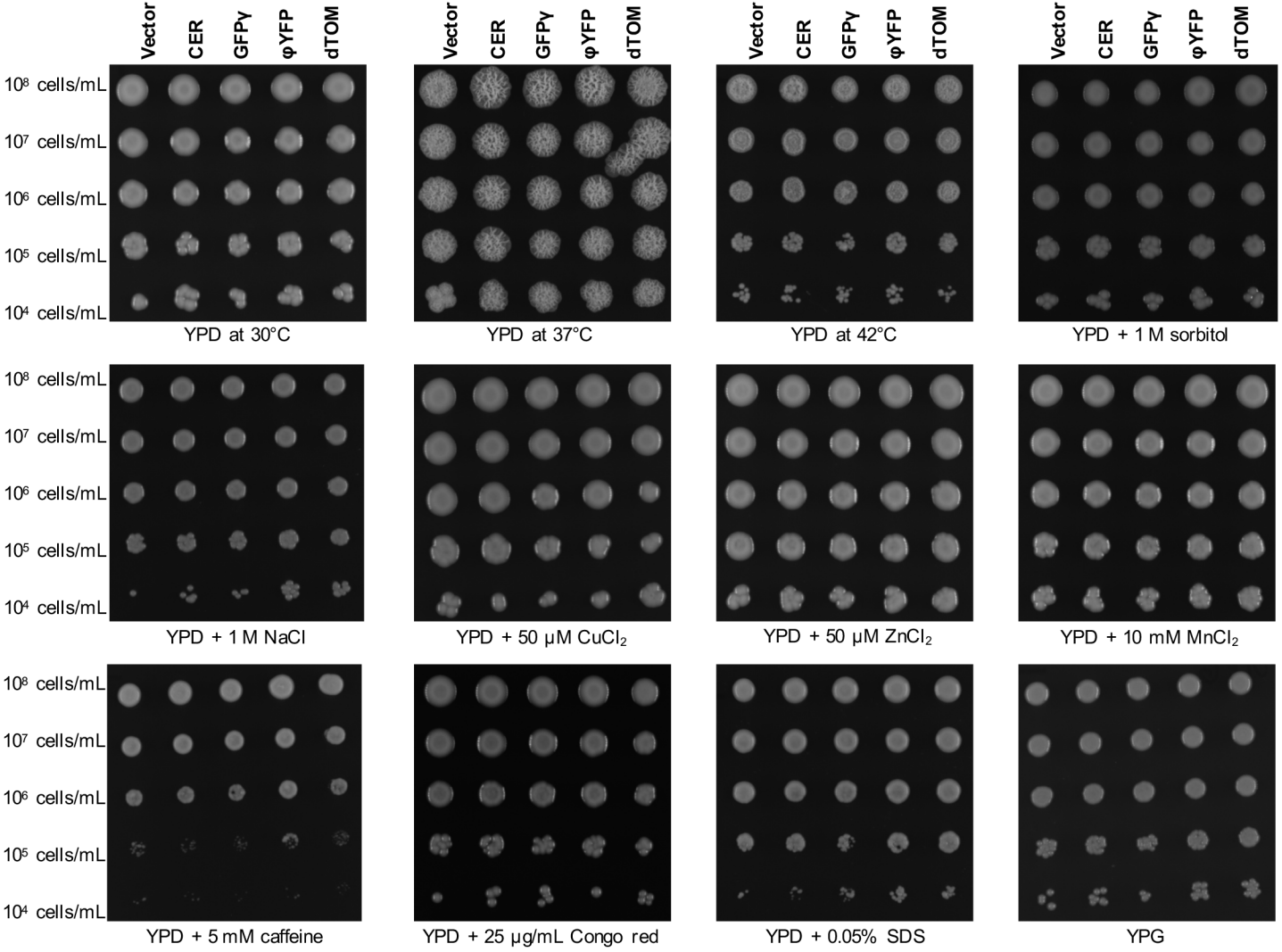

Supplement: FIG S2 [file sph005172377sf2.pdf]

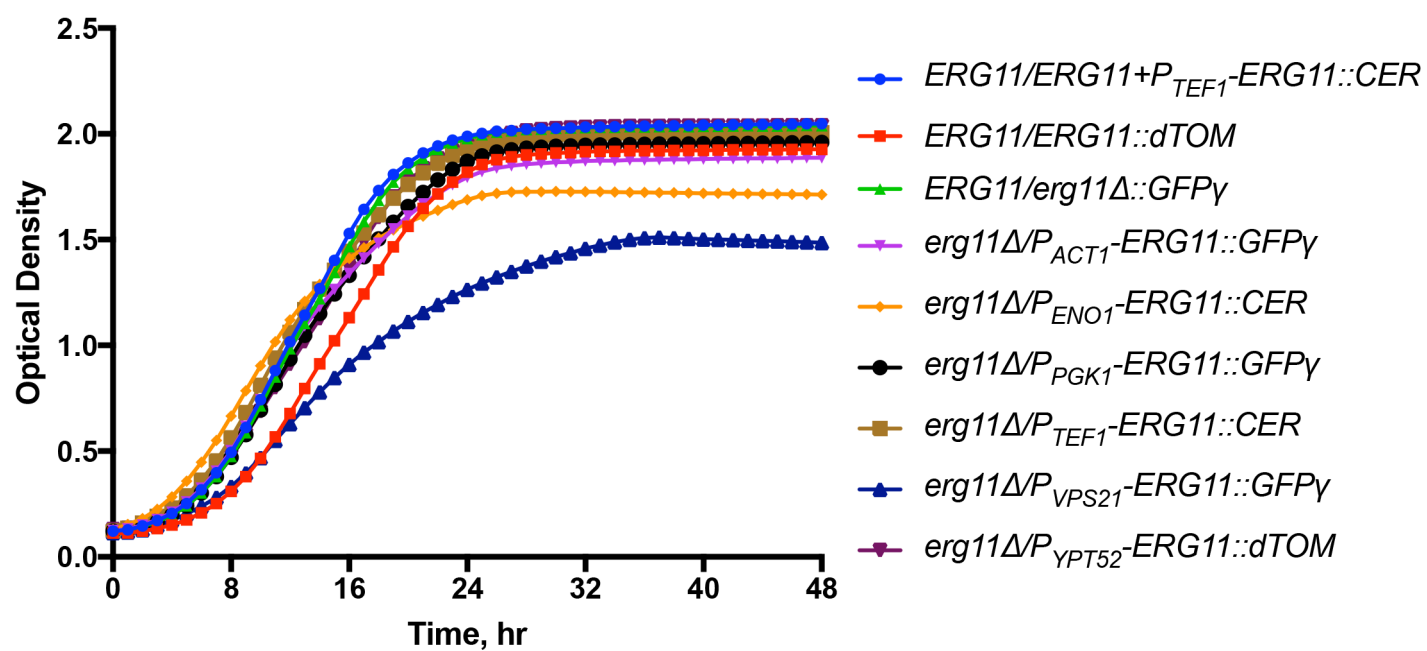

Supplement: FIG S3 [file sph005172377sf3.pdf]

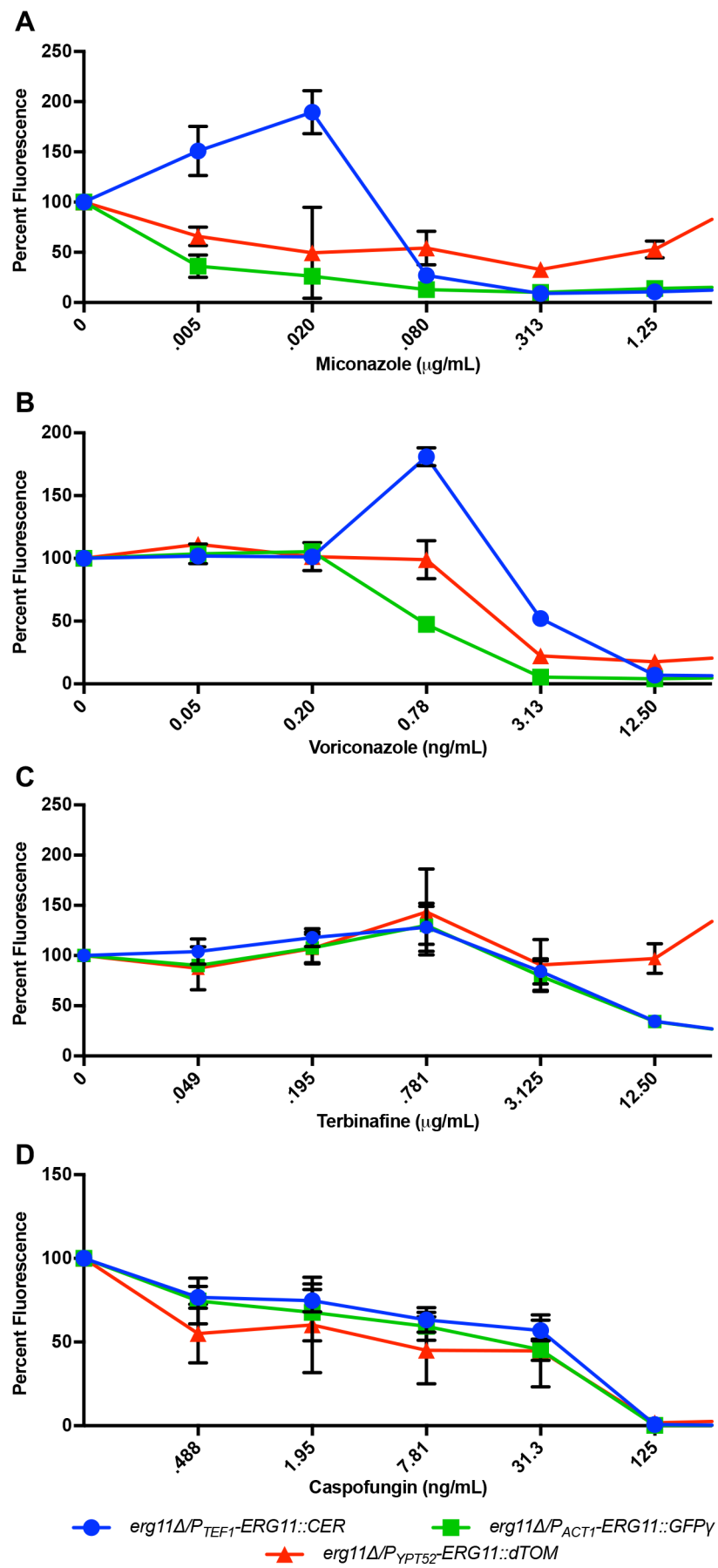

Supplement: FIG S4 [file sph005172377sf4.pdf]

**A****NCC Library at 5  $\mu$ M with Erg11 Expression Pool**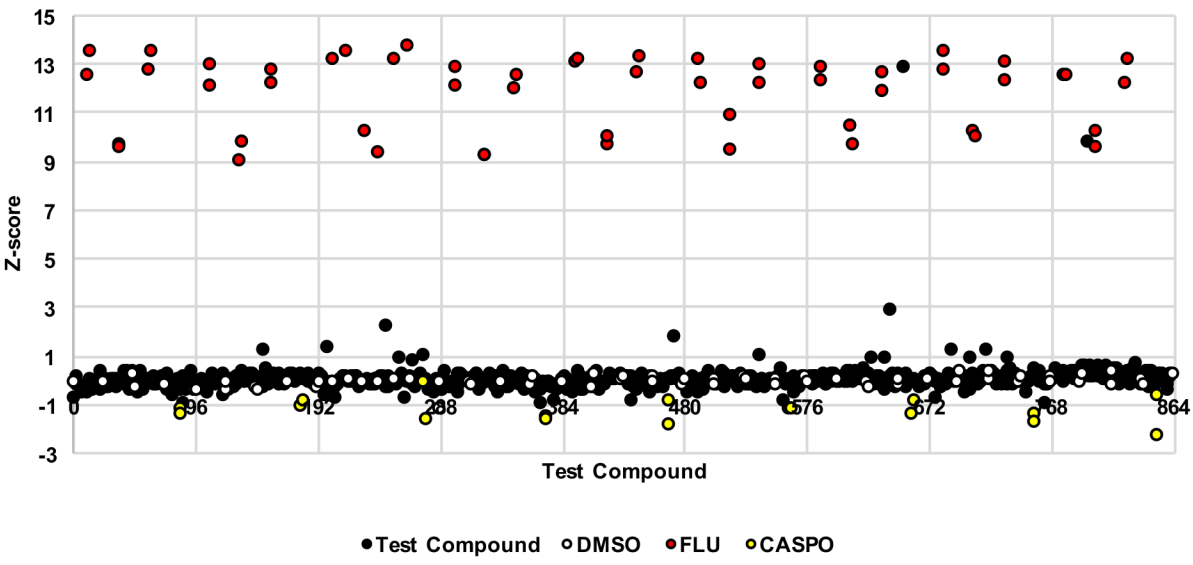**B****NCC Library at 5  $\mu$ M with Dfr1 Expression Pool**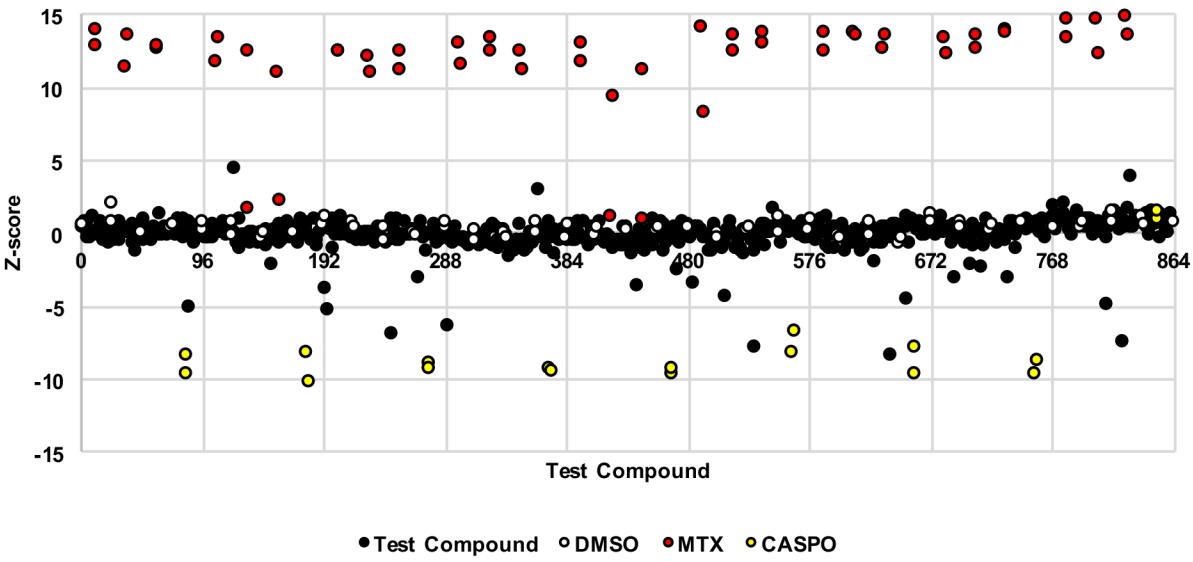

Supplement: FIG S5 [file sph005172377sf5.pdf]
