# Supplementary material for: Target Abundance-Based Fitness Screening (TAFiS) Facilitates Rapid Identification of Target-Specific and Physiologically Active Chemical Probes
Source: mSphere. 2017 Oct 4;2(5):e00379-17. doi: 10.1128/mSphere.00379-17 (PMC5628291; doi:10.1128/mSphere.00379-17)
Supplement: TEXT S1 [file sph005172377s6.docx]

**Supplemental materials and methods:**

Plasmid construction. To construct the expression vector pKE4, 565 bp of the C. albicans TEF1 promoter (*P_TEF1_*) was amplified from strain SC5314 using primers TEF1prF-KpnI and TEF1prR-SalI, cut with KpnI and SalI, and inserted between the same sites of pKE1 to replace the ACT1 promoter. This formed an expression cassette consisting of *P_TEF1_*, a multiple cloning site (SalI-ClaI-EagI-MluI-SphI), followed by 414 bp of the *ADH1* 3’ UTR sequence. This vector harbors the *URA3* selection marker of *C. albicans*, and can be targeted for integration at and fully reconstitution the *URA3-IRO1* locus of all CAI/BWP17 derived lab strains of *C. albicans*. The pAR8 expression vector was made by amplifying the *P_TEF1_-MCS-ADH1-3’UTR* cassette from pKE4 with primers TEF1prF-SpeI and ADH1-3’UTRR-SacI, and cloning the resulting product between the SpeI and SacI sites of pRSARG4∆Spe. This expression vector can be targeted for integration at the *ARG4* locus of *C. albicans*.

GFPγ, YFP, mCherry (mCh), and dTomato (dTOM) coding sequences were amplified from pFA-GFPγ, pYFPURA3, pMG2254 and pENO1-dTom-NATr plasmids with primers engineered to incorporate SalI and MluI sites either side of the coding sequence. Each was subsequently cloned between the SalI and MluI sites of plasmids pKE4 and pAR8. The coding sequences for *tagBFP* (10), *cerulean (*CER) *(12)*, φYFP *(13)*, ZsYellow *(14)*, and mPlum (15) were codon optimized according to the codon bias of a subset of highly expressed *C. albicans* proteins using the OPTIMIZER one codon/one AA program (41). Synthetic sequences incorporating SalI and MluI sites either side of each optimized coding sequence were produced by IDTDNA, amplified from the supplied DNA template using primers AMPF1 and AMPR1, and cloned between the SalI and MluI sites of the pKE4 and pAR8 vectors.

Plasmid pAR8-ERG11 were produced by amplifying the *ERG11* ORF from SC5314 using primer pair ERG11ORFF-SalI and ERG11ORFR-MluI and cloning the resulting product between SalI and MluI sites of pAR8. Plasmid pKE3-DFR1 was made by amplifying the *DFR1* ORF from SC5314 genomic DNA with DFR1ORFF-SalI and DFR1ORFR-MluI primers, and cloning the resulting product between SalI and MluI sites of pKE3. All plasmid constructs were sequence verified to ensure the correct cloning of products and absence of unintended mutations.

To facilitate replacement of endogenous transcriptional promoters in *C. albicans*, a series of plasmids were constructed in which various *C. albicans* gene promoter sequences were cloned adjacent to the *HIS1* selection marker in pGEMHIS1. *P_TEF1_* (565 bp), *P_ENO1_* (693 bp), *P_PGK1_* (870 bp), *P_ACT1_* (1000 bp), *P_YPT52_* (711 bp), and *P_VPS21_* (689 bp) promoter sequences were amplified and cloned between the BamHI and EagI sites of pGEMHIS1.

Disruption cassette construction.

The *ERG11* gene deletion cassette was amplified using ERG11DISF and ERG11DISR with pRSARG4ΔSpeI (ARG4 selection marker) as template and transformed into BWP17. Correct integration of the gene deletion cassette was confirmed by diagnostic PCR using primers ARG4INTR2 and ERG11AMPR-KpnI as well as ARG4INTF2 and ERG11AMPF2-KpnI to confirm replacement of one *ERG11* allele with the *ARG4* selection marker. The *ERG11* promoter replacement cassettes were amplified using ERG11PRF and ERG11PRR primers with each of the pGEMHIS1 based promoter plasmids described above as template. Each was then transformed into the *ERG11/erg11∆:ARG4* heterozygote strain. Transformants were selected, and those in which the native promoter of the remaining *ERG11* allele was displaced identified by the absence of a 1398 bp product following PCR amplification with the ERG11AMPF2-KpnI and ERG11DETR primer pair. Correct insertion of the desired promoter upstream of the *ERG11* ORF was then confirmed using ERG11DETR and either TEF1prDETF, ENO1prSEQF, PGK1prDETF, ACT1prSEQF, YPT52prSEQF, or VPS21prDETF.

The *DFR1* gene deletion cassette was amplified using primer set DFR1DISF and DFR1DISR and plasmid pGEMHIS1 (HIS1 selection marker) as template and transformed into BWP17. Correct integration of the gene deletion cassette to replace one *DFR1* allele was confirmed by diagnostic PCR, using primers HIS1INTR2 and DFR1AMPR-SacI as well as HIS1INTF2 and DFR1AMPF-KpnI. To further suppress the expression of the remaining *DFR1* allele, we applied an approach known as Decreased Abundance by mRNA Perturbation (DAmP)(22) to destabilize the mRNA transcript. The 3’UTR of the remaining *DFR1* allele was targeted for disruption using a deletion cassette amplified using DFR1DMPF2 and DFR1DMPR2 with pDDB57 as template. The *DFR1/dfr1∆:HIS1* heterozygous strain was transformed with the DAmP cassette, transformants selected, and the absence of an intact *DFR1* allele confirmed by the absence of a PCR product following amplification with primer pair DFR1DETF and DFR1AMPR-SacI. Correct insertion of the DDB57 (*URA3*) cassette into the 3’ UTR of *DFR1* was also confirmed by the presence of a 968 bp PCR product using primer pair DFR1DETF and URA3INTR2.
