# Supplementary material for: Target Abundance-Based Fitness Screening (TAFiS) Facilitates Rapid Identification of Target-Specific and Physiologically Active Chemical Probes
Source: mSphere. 2017 Oct 4;2(5):e00379-17. doi: 10.1128/mSphere.00379-17 (PMC5628291; doi:10.1128/mSphere.00379-17)
Supplement: TABLE S1 [file sph005172377st7.docx]

| ***Primer*** | ***Sequence 5’→3’**** |
| --- | --- |
| ACT1prF-BamHI | TCAGGATCCCCAGCCTCGTTTATAATAAACTTAGTC |
| ACT1prR-EagI | TCACGGCCGTTTGAATGATTATATTTTTTTAATATTAATATCGAG |
| ACT1prSEQF | CACCAAGATTTATTGCCAACG |
| ADH1-3’UTRR-SacI | TCATCAGAGCTCGAAAACTTGAAACTTGAAAACACC |
| AMPF1 | TGCTGAAGCTTCTTTGAGTGG |
| AMPF2 | TTCAATATTCAATGGATGAGTCCCGGCCGGTCGAC |
| AMPR1 | AGCAAAGAACATGGCAGCACC |
| AMPR2 | CACTCCCATCATGATCATGATTACCGACGCGT |
| ARGINTF2 | AAGCTAGTGTGGAAAGAAGAG |
| ARGINTR2 | AATGACTGAATTATGTCGGTC |
| DFR1AMPF-KpnI | TCAGGTACCTTGAGTTGTGGACTTAGTGGG |
| DFR1AMPR-SacI | TCAGAGCTCCGACTTTGGAGAGAGAACAGC |
| DFR1DETF | GACGTCACCACCAGAACAACG |
| DFR1DISF | TTCTTGTTTGATTGAAAAAAAACTTTCACCACTAATCTAGAACTTCAGGAATAGACAACAGTTTTCCCAGTCACGACGTT |
| DFR1DISR | TTATTTTCTTGTCCATAGCGTATAATTATAGGTAAAATCACCTTCCTTGATATCGTCTTTGTGGAATTGTGAGCGGATA |
| DFR1DMPF2 | TATTAGAAGACGATATCAAGGAAGGTGATTTTACCTATAATTATACGCTATGGACAAGAAAATAATGTGGAATTGTGAGCGGATA |
| DFR1DMPR2 | AACATCRCGAATGAAGGTGTTTGAATAAGTTATTCAATGGCCCTCTTTTTTTGTTTGTATATTTTGTTTTCCCAGTCACGACGTT |
| DFR1ORFF-SalI | TCAGTCGACAGAACTTCACGAATAGACAAC |
| DFR1ORFR-MluI | TCAACGCGTATAAGTTATTCAATGGCCCTC |
| ENO1prF2-BamHI | TCAGGTACCGTTACAATTGAGATGGAAAGCG |
| ENO1prR-EagI | TCAGTCGACTGTTGTAATATTCCTGAATTATC |
| ENO1prSEQF | CTATTTCTTTCCTTTTCTCCC |
| ERG11AMPF2-KpnI | TCAGGTAACAATGAAAATGTCGTGGGGA |
| ERG11AMPR-KpnI | TCAGGTACCACGCGTTTGGGTAGTAATTCTGTTGGC |
| ERG11DETR | TAAGGAATCAATTAAATCACG |
| ERG11DISF | TTCTTTCCATATTACTTGTCTTCTTTTTATTATATATATAAGTTTCTTTTCAAGAAGATCATAACTCAATGTTTTCCCAGTCACGACGTT |
| ERG11DISR | TGTGTTAATCCAACTAAGTAACAAAATGAAAACAATCTGAACACTGAATCGAAAGAAAGTTGCCGTTTTATGTGGAATTGTGAGCGGATA |
| ERG11ORFF-SalI | TCAGTCGACATGGCTATTGTTGAAACTGTC |
| ERG11ORFR-MluI | TCAACGCGTTGAATCGAAAGAAAGTTGCCG |
| ERG11PRF | TGTGTAGAATTTGATAAAGAGAAAAAAGAAATATTGGGTTTTGCTTGTATTCAATATCGTACCCGAGTTGAACTCCCTTATGGTGC |
| ERG11PRR | TGTTGTGTAACACTAAGGGACAAAAAATAATTAATGCCATCAATGACAGTTTCAACAATAGCCATACGTCGCATGCTCCCGGCCG |
| HIS1INTF2 | ACTGTATCCTCTTCTGTCCCC |
| HIS1INTR2 | CGACCATATGGGAGAGCTCCC |
| PGK1prDETF | AGCTATAGGGAAGGCAATTGG |
| PGK1prF-BamHI | TCAGGATCCGCAATAGTTCCATCTGGCTCG |
| PGK1prR-EagII | TCACGGCCGTTTGATAGTTATTCTTCTGCA |
| TEF1prDETF | ATGGATCAGCTGTGCTTGTGG |
| TEF1prF-BamHI | TCAGGATCCTGCAAATCTGTTTGCTGATGG |
| TEF1prF-KpnI | TCAGGTACCTGCAAATCTGTTTGCTGATGG |
| TEF1prF-SpeI | TCAACTAGTTGCAAATCTGTTTGCTGATGG |
| TEF1prR-EagI | TCACGGCCGGATTGATTATGACTATAATGTG |
| TEF1prR-KpnI | TCAGGTACCGATTGATTATGACTATAATGTG |
| URA3INTR2 | CAAGAAGAAGGATTTGATTGGC |
| VPS21prDETF | ATCAAGGTCGTGCCTTATCGC |
| VPS21prF-BamHI | TCAGGATCCCCGAAGAAAAGGGGAAGGAGA |
| VPS21prR-EagI | TCACGGCCGTGCTAGTATAGTATTTTGGCTC |
| YPT52prF-BamHI | TCAGGATCCAATCTTTACAACCATTGTTCG |
| YPT52prR-EagI | TCACGGCCGGAAAATAAATTTAACTTGGAATA |
| YPT52prSEQF | TATATTGTATACCTTCACCTC |
