# Supplementary material for: Target Abundance-Based Fitness Screening (TAFiS) Facilitates Rapid Identification of Target-Specific and Physiologically Active Chemical Probes
Source: mSphere. 2017 Oct 4;2(5):e00379-17. doi: 10.1128/mSphere.00379-17 (PMC5628291; doi:10.1128/mSphere.00379-17)
Supplement: TABLE S2 [file sph005172377st8.docx]

| ***Synthetic sequence*** | ***Sequence 5’→3’**** |
| --- | --- |
| *CatagBFP* | **TGCTGAAGCTTCTTTGAGTGG***TTCAATATTCAATGGATGAGTCCCGGCCGGTCGAC*ATGTCTGAAGAATTGATTAAAGAAAACATGCACATGAAATTGTACATGGAAGGTACTGTTGATAACCACCACTTCAAATGTACTTCTGAAGGTGAAGGTAAACCATACGAAGGTACTCAAACTATGAGAATTAAAGTTGTTGAAGGTGGTCCATTGCCATTCGCTTTCGATATTTTGGCTACTTCTTTCTTGTACGGTTCTAAAACTTTCATTAACCACACTCAAGTTATTCCAGATTTCTTCAAACAATCTTTCCCAGAAGGTTTCACTTGGGAAAGAGTTACTACTTACGAAGATGGTGGTGTTTTGACTGCTACTCAAGATACTTCTTTGCAAGATGGTTGTTTGATTTACAACGTTAAAATTAGAGGTGTTAACTTCACTTCTAACGGTCCAGTTATGCAAAAAAAAACTTTGGGTTGGGAAGCTTTCACTGAAACTTTGTACCCAGCTGATGGTGGTTTGGAAGGTAGAAACGATATGGCTTTGAAATTGGTTGGTGGTTCTCACTTGATTGCTAACATTAAAACTACTTACAGATCTAAAAAACCAGCTAAAAACTTGAAAATGCCAGGTGTTTACTACGTTGATTACAGATTGGAAAGAATTAAAGAAGCTAACAACGAAACTTACGTTGAACAACACGAAGTTGCTGTTGCTAGATACTGTGATTTGCCATCTAAATTGGGTCACAAATTGAACTAA*ACGCGTCGGTAATGATCATGATGGGAGTG***GGTGCTGCCATGTTCTTTGCT** |
| *CaCER* | **TGCTGAAGCTTCTTTGAGTGG***TTCAATATTCAATGGATGAGTCCCGGCCGGTCGAC*ATGGTTTCTAAAGGTGAAGAATTGTTCACTGGTGTTGTTCCAATTTTGGTTGAATTGGATGGTGATGTTAACGGTCACAGATTCTCTGTTTCTGGTGAAGGTGAAGGTGATGCTACTTACGGTAAATTGACTTTGAAATTCATTTGTACTACTGGTAAATTGCCAGTTCCATGGCCAACTTTGGTTACTACTTTGACTTGGGGTGTTCAATGTTTCGCTAGATACCCAGATCACATGAAACAACACGATTTCTTCAAATCTGCTATGCCAGAAGGTTACGTTCAAGAAAGAACTATTTTCTTCAAAGATGATGGTAACTACAAAACTAGAGCTGAAGTTAAATTCGAAGGTGATACTTTGGTTAACAGAATTGAATTGAAAGGTATTGATTTCAAAGAAGATGGTAACATTTTGGGTCACAAATTGGAATACAACGCTATTTCTGATAACGTTTACATTACTGCTGATAAACAAAAAAACGGTATTAAAGCTCACTTCAAAATTAGACACAACATTGAAGATGGTTCTGTTCAATTGGCTGATCACTACCAACAAAACACTCCAATTGGTGATGGTCCAGTTTTGTTGCCAGATAACCACTACTTGTCTACTCAATCTGCTTTGTCTAAAGATCCAAACGAAAAAAGAGATCACATGGTTTTGTTGGAATTCGTTACTGCTGCTGGTATTACTTTGGGTATGGATGAATTGTACAAATAG*ACGCGTCGGTAATGATCATGATGGGAGTG***GGTGCTGCCATGTTCTTTGCT** |
| *CaφYFP* | **TGCTGAAGCTTCTTTGAGTGG***TTCAATATTCAATGGATGAGTCCCGGCCGGTCGAC*ATGTCTTCTGGTGCTTTGTTGTTCCACGGTAAAATTCCATACGTTGTTGAAATGGAAGGTAACGTTGATGGTCACACTTTCTCTATTAGAGGTAAAGGTTACGGTGATGCTTCTGTTGGTAAAGTTGATGCTCAATTCATTTGTACTACTGGTGATGTTCCAGTTCCATGGTCTACTTTGGTTACTACTTTGACTTACGGTGCTCAATGTTTCGCTAAATACGGTCCAGAATTGAAAGATTTCTACAAATCTTGTATGCCAGATGGTTACGTTCAAGAAAGAACTATTACTTTCGAAGGTGATGGTAACTTCAAAACTAGAGCTGAAGTTACTTTCGAAAACGGTTCTGTTTACAACAGAGTTAAATTGAACGGTCAAGGTTTCAAAAAAGATGGTCACGTTTTGGGTAAAAACTTGGAATTCAACTTCACTCCACACTGTTTGTACATTTGGGGTGATCAAGCTAACCACGGTTTGAAATCTGCTTTCAAAATTTGTCACGAAATTACTGGTTCTAAAGGTGATTTCATTGTTGCTGATCACACTCAAATGAACACTCCAATTGGTGGTGGTCCAGTTCACGTTCCAGAATACCACCACATGTCTTACCACGTTAAATTGTCTAAAGATGTTACTGATCACAGAGATAACATGTCTTTGAAAGAAACTGTTAGAGCTGTTGATTGTAGAAAAACTTACTTGTGAA*ACGCGTCGGTAATGATCATGATGGGAGTG***GGTGCTGCCATGTTCTTTGCT** |
| *CaZsYellow* | **TGCTGAAGCTTCTTTGAGTGG***TTCAATATTCAATGGATGAGTCCCGGCCGGTCGAC*ATGGCTCACTCTAAACACGGTTTAAAAGAAGAAATGACTATGAAATATCACATGGAAGGTTGTGTTAACGGTCACAAATTCGTTATTACTGGTGAAGGTATTGGTTATCCATTCAAAGGTAAACAAACTATTAACTTATGTGTTATTGAAGGTGGTCCATTACCATTCTCTGAAGATATTTTATCTGCTGGTTTCAAATATGGTGATCGTATTTTCACTGAATATCCACAAGATATTGTTGATTATTTCAAAAACTCTTGTCCAGCTGGTTATACTTGGGGTCGTTCTTTCTTATTCGAAGATGGTGCTGTTTGTATTTGTAACGTTGATATTACTGTTTCTGTTAAAGAAAACTGTATTTATCACAAATCTATTTTCAACGGTGTTAACTTCCCAGCTGATGGTCCAGTTATGAAAAAAATGACTACTAACTGGGAAGCTTCTTGTGAAAAAATTATGCCAGTTCCAAAACAAGGTATTTTAAAAGGTGATGTTTCTATGTATTTATTATTAAAAGATGGTGGTCGTTATCGTTGTCAATTCGATACTGTTTATAAAGCTAAATCTGTTCCATCTAAAATGCCAGAATGGCACTTCATTCAACACAAATTATTACGTGAAGATCGTTCTGATGCTAAAAACCAAAAATGGCAATTAACTGAACACGCTATTGCTTTCCCATCTGCTTTAGCTTGA*ACGCGTCGGTAATGATCATGATGGGAGTG***GGTGCTGCCATGTTCTTTGCT** |
| *CamPLUM* | **TGCTGAAGCTTCTTTGAGTGG***TTCAATATTCAATGGATGAGTCCCGGCCGGTCGAC*ATGGTTTCTAAAGGTGAAGAAGTTATTAAAGAATTCATGAGATTCAAAGAACACATGGAAGGTTCTGTTAACGGTCACGAATTCGAAATTGAAGGTGAAGGTGAAGGTAGACCATACGAAGGTACTCAAACTGCTAGATTGAAAGTTACTAAAGGTGGTCCATTGCCATTCGCTTGGGATATTTTGTCTCCACAAATTATGTACGGTTCTAAAGCTTACGTTAAACACCCAGCTGATATTCCAGATTACTTGAAATTGTCTTTCCCAGAAGGTTTCAAATGGGAAAGAGTTATGAACTTCGAAGATGGTGGTGTTGTTACTGTTACTCAAGATTCTTCTTTGCAAGATGGTGAATTCATTTACAAAGTTAAAGTTAGAGGTACTAACTTCCCATCTGATGGTCCAGTTATGCAAAAAAAAACTATGGGTTGGGAAGCTTCTTCTGAAAGAATGTACCCAGAAGATGGTGCTTTGAAAGGTGAAATGAAAATGAGATTGAGATTGAAAGATGGTGGTCACTACGATGCTGAAGTTAAAACTACTTACATGGCTAAAAAACCAGTTCAATTGCCAGGTGCTTACAAAACTGATATTAAATTGGATATTACTTCTCACAACGAAGATTACACTATTGTTGAACAATACGAAAGAGCTGAAGGTAGACACTCTACTGGTGCT*ACGCGTCGGTAATGATCATGATGGGAGTG***GGTGCTGCCATGTTCTTTGCT** |
